# Relationships between intensity, duration, cumulative dose, and timing of smoking with age at menopause: A pooled analysis of individual data from 17 observational studies

**DOI:** 10.1371/journal.pmed.1002704

**Published:** 2018-11-27

**Authors:** Dongshan Zhu, Hsin-Fang Chung, Nirmala Pandeya, Annette J. Dobson, Janet E. Cade, Darren C. Greenwood, Sybil L. Crawford, Nancy E. Avis, Ellen B. Gold, Ellen S. Mitchell, Nancy F. Woods, Debra Anderson, Daniel E. Brown, Lynnette L. Sievert, Eric J. Brunner, Diana Kuh, Rebecca Hardy, Kunihiko Hayashi, Jung Su Lee, Hideki Mizunuma, Graham G. Giles, Fiona Bruinsma, Therese Tillin, Mette Kildevæld Simonsen, Hans-Olov Adami, Elisabete Weiderpass, Marianne Canonico, Marie-Laure Ancelin, Panayotes Demakakos, Gita D. Mishra

**Affiliations:** 1 The University of Queensland, School of Public Health, Brisbane, Queensland, Australia; 2 Department of Population Health, QIMR Berghofer Medical Research Institute, Brisbane, Queensland, Australia; 3 Nutritional Epidemiology Group, School of Food Science and Nutrition, University of Leeds, Leeds, United Kingdom; 4 Department of Medicine, University of Massachusetts Medical School, Worcester, Massachusetts, United States of America; 5 Department of Social Sciences and Health Policy, Wake Forest School of Medicine, Winston-Salem, North Carolina; 6 Department of Public Health Sciences, University of California, Davis School of Medicine, California, United States of America; 7 Family and Child Nursing, School of Nursing, University of Washington, Seattle, Washington, United States of America; 8 Biobehavioral Nursing and Health Systems, School of Nursing, University of Washington, Seattle, Washington, United States of America; 9 Menzies Health Institute Queensland, Griffith University, Gold Coast, Queensland, Australia; 10 Department of Anthropology, University of Hawaii, Hilo, Hawaii, United States of America; 11 Department of Anthropology, UMass Amherst, Amherst, Massachusetts, United States of America; 12 Department of Epidemiology and Public Health, University College London, London, United Kingdom; 13 Medical Research Council Unit for Lifelong Health and Ageing at UCL, London, United Kingdom; 14 School of Health Sciences, Gunma University, Maebashi City, Gunma, Japan; 15 Department of Public Health, Graduate School of Medicine, The University of Tokyo, Tokyo, Japan; 16 Fukushima Medical Center for Children and Women, Fukushima Medical University, Fukushima, Japan; 17 Cancer Epidemiology and Intelligence Division, Cancer Council Victoria, Melbourne, Victoria, Australia; 18 Centre for Epidemiology and Biostatistics, Melbourne School of Population and Global Health, The University of Melbourne, Melbourne, Victoria, Australia; 19 Institute of Cardiovascular Science, University College London, London, United Kingdom; 20 UcDiakonissen and Parker Institute, Frederiksberg, Denmark; 21 Department of Medical Epidemiology and Biostatistics, Karolinska Institutet, Stockholm, Sweden; 22 Clinical Effectiveness Research Group, Institute of Health and Society, University of Oslo, Oslo, Norway; 23 Genetic Epidemiology Group, Folkhälsan Research Center, Helsinki, Finland; 24 Department of Community Medicine, Faculty of Health Sciences, University of Tromsø, The Arctic University of Norway, Tromsø, Norway; 25 Department of Research, Cancer Registry of Norway, Institute of Population-Based Cancer Research, Oslo, Norway; 26 Paris-Saclay University, Paris-South University, UVSQ, Center for Research in Epidemiology and Population Health, INSERM, France; 27 INSERM, University Montpellier, Neuropsychiatry: Epidemiological and Clinical Research, Montpellier, France; Stanford University, UNITED STATES

## Abstract

**Background:**

Cigarette smoking is associated with earlier menopause, but the impact of being a former smoker and any dose-response relationships on the degree of smoking and age at menopause have been less clear. If the toxic impact of cigarette smoking on ovarian function is irreversible, we hypothesized that even former smokers might experience earlier menopause, and variations in intensity, duration, cumulative dose, and age at start/quit of smoking might have varying impacts on the risk of experiencing earlier menopause.

**Methods and findings:**

A total of 207,231 and 27,580 postmenopausal women were included in the cross-sectional and prospective analyses, respectively. They were from 17 studies in 7 countries (Australia, Denmark, France, Japan, Sweden, United Kingdom, United States) that contributed data to the International collaboration for a Life course Approach to reproductive health and Chronic disease Events (InterLACE). Information on smoking status, cigarettes smoked per day (intensity), smoking duration, pack-years (cumulative dose), age started, and years since quitting smoking was collected at baseline. We used multinomial logistic regression models to estimate multivariable relative risk ratios (RRRs) and 95% confidence intervals (CIs) for the associations between each smoking measure and categorised age at menopause (<40 (premature), 40–44 (early), 45–49, 50–51 (reference), and ≥52 years). The association with current and former smokers was analysed separately. Sensitivity analyses and two-step meta-analyses were also conducted to test the results. The Bayesian information criterion (BIC) was used to compare the fit of the models of smoking measures.

Overall, 1.9% and 7.3% of women experienced premature and early menopause, respectively. Compared with never smokers, current smokers had around twice the risk of experiencing premature (RRR 2.05; 95% CI 1.73–2.44) (*p* < 0.001) and early menopause (1.80; 1.66–1.95) (*p* < 0.001). The corresponding RRRs in former smokers were attenuated to 1.13 (1.04–1.23; *p* = 0.006) and 1.15 (1.05–1.27; *p* = 0.005). In both current and former smokers, dose-response relationships were observed, i.e., higher intensity, longer duration, higher cumulative dose, earlier age at start smoking, and shorter time since quitting smoking were significantly associated with higher risk of premature and early menopause, as well as earlier menopause at 45–49 years. Duration of smoking was a strong predictor of age at natural menopause. Among current smokers with duration of 15–20 years, the risk was markedly higher for premature (15.58; 11.29–19.86; *p* < 0.001) and early (6.55; 5.04–8.52; *p* < 0.001) menopause. Also, current smokers with 11–15 pack-years had over 4-fold (4.35; 2.78–5.92; *p* < 0.001) and 3-fold (3.01; 2.15–4.21; *p* < 0.001) risk of premature and early menopause, respectively. Smokers who had quit smoking for more than 10 years had similar risk as never smokers (1.04; 0.98–1.10; *p* = 0.176). A limitation of the study is the measurement errors that may have arisen due to recall bias.

**Conclusions:**

The probability of earlier menopause is positively associated with intensity, duration, cumulative dose, and earlier initiation of smoking. Smoking duration is a much stronger predictor of premature and early menopause than others. Our findings highlight the clear benefits for women of early smoking cessation to lower their excess risk of earlier menopause.

## Abstract 2

In a pooled analysis of 17 observational studies, Gita Mishra and colleagues investigate whether former, as well as current, smokers are at risk of early menopause, and whether smoking duration and intensity are also associated with early menopause.

## Author summary

**Why was this study done?:**

- Current smoking is associated with earlier age at natural menopause.
- The impact of former smoking on age at natural menopause is inconclusive, and the dose-response relationships between the degree of smoking (including intensity and duration) and age at natural menopause have been less clear.

**What did the researchers do and find?:**

- We pooled individual-level data from 17 observational studies that contributed to the International collaboration for a Life course Approach to reproductive health and Chronic disease Events (InterLACE) consortium. A total of 207,231 and 27,580 postmenopausal women were included in the cross-sectional and prospective analyses, respectively.
- We examined the associations between smoking measures (smoking status, intensity, duration, cumulative dose, and timing of cigarette smoking) and age at natural menopause.
- Compared with never smokers, former smokers had around 15% higher risk of premature and early menopause, but this was much lower than that of current smokers, who had double the risk when compared to never smokers.
- Early menopause is positively associated with intensity, duration, cumulative dose, and earlier initiation of smoking in both current and former smokers. Duration of smoking was a strong predictor of early menopause.

**What do these findings mean?:**

- Our findings highlight the clear benefits for women of early smoking cessation to lower their excess risk of earlier menopause, thereby reducing the increased risks of chronic diseases (e.g., cardiovascular disease and osteoporosis) associated with both smoking and earlier menopause.

## Introduction

Natural menopause is defined as when a woman has had no menstrual periods for 12 consecutive months that did not result from interventions (such as bilateral oophorectomy, hysterectomy, chemotherapy, or radiotherapy). It typically occurs between 49 and 52 years of age, with a median age of 51.4 years in high-income countries [1–3]. Approximately 5% of women report early menopause [4,5], occurring between ages 40 and 45 years, whereas premature menopause—also termed premature ovarian failure (POF)—occurs before age 40 years [5] in about 1% of women [6,7]. Women with early or premature menopause are at increased risk of morbidity and mortality in later life, including cardiovascular disease [8,9], osteoporosis [9], and type 2 diabetes [10,11].

In addition to genetic factors [5], socioeconomic status, body weight, cigarette smoking, race/ethnicity, age at menarche, and nulliparity influence age at menopause [12–15]. Cigarette smoking is the most established factor and leads to having menopause by almost one year earlier [3,15,16], but important knowledge gaps remain. A recent systematic review of 109 studies concluded that although current smoking is associated with earlier age at menopause, evidence for the impact of being a former smoker was inconclusive. In addition, dose-response relationships on the degree of smoking and age at menopause have been less clear [16], and few studies have examined the relationship between age at start [17,18] or quit of smoking [19,20] and age at menopause. If the hypothesized toxic impact of cigarette smoking on ovarian function is irreversible [21], even former smokers may experience earlier menopause, and variations in intensity, duration, cumulative dose, as well as age at start/quit of smoking might have varying impacts on the risk of experiencing earlier menopause.

To investigate these issues, we analysed individual-level data from more than 200,000 postmenopausal women in 17 studies that contributed data to the International collaboration for a Life course Approach to reproductive health and Chronic disease Events (InterLACE) [22,23]. Dose-response relationships were investigated between the multiple aspects of smoking and age at menopause in both cross-sectional and prospective analyses.

## Methods

### Participants

InterLACE has pooled individual-level data on reproductive health and chronic diseases from over 500,000 women from 25 observational studies across 10 countries. Most studies are prospective and collected survey data on key reproductive, sociodemographic, and lifestyle factors, as well as disease outcomes. Participants in each of the included studies were recruited and provided consent according to the approved protocols of the Institutional Review Board or Human Research Ethics Committee at each relevant institution. More detailed descriptions of the InterLACE consortium have been published previously [22,23]. For the present analyses, 17 studies of InterLACE (Table 1) provided information on women’s smoking status at the baseline survey and age at natural menopause. Of these, 14 collected longitudinal data, while the remaining 3 provided only cross-sectional information. As the use of hormone therapy (HT) and oral contraceptive pills (OCPs) precludes accurate definition of menopausal status, hormone users were excluded from the analyses unless natural menopause was specifically reported. The cross-sectional analysis was based on 207,231 postmenopausal women with information on age at natural menopause (i.e., women who experienced surgical menopause were excluded), smoking status, and key covariates at baseline, BMI, years of education, and race/ethnicity/region. The prospective analysis was based on 27,580 women who experienced menopause after the baseline survey. This study is reported as per the Strengthening the Reporting of Observational Studies in Epidemiology (STROBE) guidelines (S1 Text).

**Table 1:** Characteristics of individual studies in the InterLACE consortium for cross-sectional analysis*.

Study	Country	*N*	Age in years at baseline		Age in years at last follow-up	Women's year of birth (%)			
			Mean (SD)	Median (Q1, Q3)	Mean (SD)	<1930	1930–1939	1940–1949	1950+
ALSWH	Australia	7,353	47.6 (1.4)	47.6 (46.4, 48.9)	63.1 (3.4)	-	-	75	25
HOW	Australia	338	54.8 (2.7)	55.0 (53.0, 57.0)	62.5 (4.0)	-	-	87.9	12.1
MCCS	Australia	12,876	58.7 (7.2)	59.5 (53.6, 64.6)	67.9 (7.6)	35.6	42.6	19.8	2
DNC	Denmark	8,868	59.6 (7.8)	58.0 (54.0, 64.0)	69.8 (8.8)	29.2	51.3	19.4	-
French 3C	France	4,253	74.5 (5.7)	73.9 (69.9, 78.3)	74.5 (5.7)	72.2	27.8	-	-
JNHS	Japan	5,331	54.7 (4.0)	55.0 (52.0, 57.0)	54.7 (4.0)	-	1.6	63.9	34.5
WLH	Sweden	11,071	45.0 (3.6)	46.0 (43.0, 48.0)	55.8 (3.8)	-	-	77	23
MRC NSHD^†^	UK	732	47.0	47.0	53.8	-	-	100	-
NCDS^†^	UK	2,542	50.0	50.0	54.8	-	-	-	100
ELSA	UK	2,967	60.4 (10.0)	59.0 (52.0, 68.0)	70.8 (9.6)	21.2	28.5	37.3	13.1
UKWCS	UK	8,465	58.1 (7.3)	58.0 (52.8, 63.5)	60.9 (7.3)	13.5	42.4	39	5.1
WHITEHALL	UK	1,647	46.5 (5.9)	47.0 (41.0, 52.0)	64.1 (6.4)	-	49.9	43.8	6.2
SABRE	UK	453	56.5 (5.0)	57.0 (53.0, 60.0)	63.4 (9.8)	19.9	67.1	13	-
UK Biobank	UK	138,014	59.7 (5.6)	60.0 (56.0, 64.0)	59.7 (5.6)	-	4.1	55.8	40.1
HILO	USA	306	55.8 (4.8)	55.6 (52.8, 59.4)	55.8 (4.8)	-	-	52.9	46.7
SWAN	USA	1,907	46.5 (2.6)	46.0 (44.0, 48.0)	56.0 (2.9)	-	-	49.1	50.9
Seattle Middle Women's Health Study (SMWHS	USA	108	42.0 (4.4)	41.9 (38.3, 45.0)	49.9 (3.8)	-	2.8	52.8	44.4
All		207,231	58.0 (7.7)	59.0 (53.0, 63.9)	60.9 (7.1)	5.8	10.9	51.2	32.1

*In the cross-sectional analysis, women who had complete information on age at menopause, smoking status, BMI, education level, and race/ethnicity/region at baseline were included.

^†^NSHD (1946 British Birth Cohort) and NCDS (1958 British Birth Cohort) first collected information on women’s health in 1993 (aged 47) and 2008 (aged 50), respectively, so we used age 47 and age 50 as the baseline age for the current study.

**Abbreviations:** ALSWH, Australian Longitudinal Study on Women’s Health; DNC, Danish Nurse Cohort Study; ELSA, English Longitudinal Study of Ageing; French 3C, French Three-City Study; HILO, Hilo Women’s Healthy Study; HOW, Healthy Ageing of Women Study; InterLACE, International Collaboration for a Life Course Approach to Reproductive Health and Chronic Disease Events; JNHS, Japan Nurses’ Health Study; MCCS, Melbourne Collaborative Cohort Study; MRC, Medical Research Council; NSHD, National Survey of Health and Development; NCDS, National Child Development Study; Q1, the first quartile; Q3, the third quartile; SABRE, Southall And Brent Revisited; SMWHS, Seattle Middle Women's Health Study; SWAN, Study of Women's Health Across the Nation; UKWCS, UK Women's Cohort Study; WHITEHALL, Whitehall II study; WLH, Women’s Lifestyle and Health Study.

### Outcome and exposure variables

Age at natural menopause was the outcome variable and was self-reported. In the cross-sectional analyses, age at menopause was categorised as <40 years (premature menopause), 40–44 (early menopause), 45–49, 50–51 (reference category), and 52 years and above, while in the prospective analyses—due to the insufficient number of women with premature menopause—age at menopause was classified into 4 categories by combining the premature and early menopause groups as <45 years.

Information on smoking status, intensity, duration, and timing of cigarette smoking was collected through self-reported questionnaires. Smoking status was categorised as current, former, or never. Current smokers were defined as women who started smoking before menopause and were regular smokers at baseline or who experienced menopause before they quit smoking. Former smokers were defined as women who experienced menopause after they quit smoking at least 1 year. Smoking intensity was defined as the average number of cigarettes smoked per day categorised as 1–9, 10–19, and 20 cigarettes or more. Among former smokers, duration was defined as the difference between the reported age at starting and quitting and, for current smokers, the difference between the age at starting and age at menopause. Therefore, by definition of duration in current smokers, women with later menopause would tend to have a longer duration. In order to observe the association between smoking duration and earlier menopause, we focused on short duration of smoking (similar reason for categories of pack-years and years since quitting smoking). To determine appropriate categories for duration of smoking, we used a fractional-polynomial model to plot the shape of the relationship between smoking duration (in all current smokers) and age at menopause. As expected, we found a U-shape, and duration of 20 years was determined as an appropriate cut-point to define a maximum duration that had an impact on age at menopause. Duration of smoking was then categorised as <10, 10–14, and 15–20 years. The cumulative dose of smoking was calculated as pack-years (the number of cigarettes smoked per day divided by 20 [assuming 20 cigarettes a pack] and multiplied by the duration of smoking) [17]. We categorized pack-years as ≤5, 6–10, and 11–15. Other smoking characteristics included age when a woman started smoking (categorised as <15, 15–19, 20 or more years of age), and years since quitting smoking (calculated by taking the difference between age at menopause and age at quitting). Inherent in the definition of years since quitting smoking, age at menopause places an upper time limit possible. Thus, in the analysis we focused on the more recent years since quitting (categorised as 1–5, 6–10, and 11–15 years).

### Covariates

Baseline BMI, years of education, race/ethnicity/region, parity, and age at menarche were used as covariates because these have been shown to be associated with age at menopause [14,15]. BMI was categorised according to World Health Organization (WHO) criteria as <18.5 kg/m^2^, 18.5 to 24.9 kg/m^2^, 25 to 29.9 kg/m^2^, and ≥30 kg/m^2^. Years of education was categorised into ≤10, 11–12, and >12 years. Race/ethnicity/region was combined into one, with 7 categories: White European, White Australian/New Zealand, White American/Canadian, Japanese, Other Asian, African American/Black, and Other. Parity was categorised as none, one child, two children, and three or more children. Age at menarche was divided into 5 categories as ≤11, 12, 13, 14, and 15 years or more.

### Statistical analyses

The data analyses for the present study were performed following a prospective research proposal (S2 Text). We performed pooled analyses of all the individual-level data. Multinomial (polytomous) logistic regression models with 5 categories of outcome for age at menopause (<40, 40–44, 45–49, 50–51, and 52 years and older) were used, with age 50–51 years at menopause as a reference group. The intensity, duration, cumulative dose, and age started smoking were analysed in combination with smoking status for current and former smokers. Never smokers were used as reference for all smoking measures. All statistical models were adjusted for BMI, education level, a combined variable for race/ethnicity/region, based on the established relations of these in the literature to the outcome. Age at menarche and parity were also potential confounders that could affect the association between smoking exposures and age at menopause. Thus, the models were additionally adjusted for both covariates but with only 14 studies included because the WHITEHALL and Southall And Brent Revisited (SABRE) studies did not collect age at menarche and Healthy Ageing of Women (HOW) study did not collect parity. Adjusted RRRs (i.e., the ratio of two relative risks, RRRs) [24] and 95% confidence intervals (CIs) for smoking measures and each category of age at menopause were estimated. The Bayesian information criterion (BIC) was used to compare the fit of the models that used each of the measures of smoking characteristic. A lower BIC implies a better fit.

#### Sensitivity analyses and two-step meta-analyses

Because the UK Biobank data contributed more than 60% of the total sample used in the cross-sectional analyses, we conducted a sensitivity analysis by excluding this study. Also, we analysed the associations of intensity, duration, age of starting smoking, and years since quitting with age at menopause by adjusting for all covariates, including the other smoking characteristics. Finally, in order to evaluate the heterogeneity among studies, in both cross-sectional and prospective analyses, we also performed study-specific regressions and random-effects meta-analysis for the associations between each level of the cigarette smoking measures and each category of menopausal age to estimate the magnitude of association.

Statistical analyses were performed by using SAS (version 9.4; SAS Institute Inc., Cary, NC) and Stata (version 14.0; Stata Corp., College Station, TX). In SAS, the SURVEYLOGISTIC procedure was used with the generalized logit link to adjust for the clustering of data within studies to obtain robust standard errors. The SGPANEL procedure was used to plot the associations. In STATA, the METAN command was used to perform meta-analysis. All statistical tests were based on the two-sided 5% level of significance.

## Results

### Baseline characteristics

The mean age at baseline was 58.0 years (range 42.0 to 74.5 years), with more than half of the women born between 1940 and 1949 (Table 1). Most women were white European (85.8%). The mean age at natural menopause was 50.2 years (SD: 4.4), and the median was 50.0 years (interquartile range: 48.0–53.0), with 57.5% of participants being never smokers, 30.7% former, and 11.8% current smokers at baseline. Current smokers had a longer duration of smoking (mean: 29.6 versus 19.4 years) and higher number of pack-years (mean: 22.9 versus 16.7 pack-years) than former smokers (Table 2).

**Table 2:** Overall baseline characteristics of women used for cross-sectional analysis (*n* = 207,231).

Characteristics	*n* (%) or mean ± SD
**Race/ethnicity/region**	
White (Australia)	14,594 (7.0)
White (Europe)	177,810 (85.8)
White (USA)	1,187 (0.6)
Japanese	5,644 (2.7)
Other Asian	2,918 (1.4)
African American/Black	2,489 (1.2)
Other	2,589 (1.3)
**Education level**	
≤10 years	99,615 (48.1)
11–12 years	24,292 (11.7)
>12 years	83,324 (40.2)
**BMI (kg/m**^**2**^**)**	
Underweight, <18.5	4,303 (2.1)
Normal, 18.5–24.9	91,690 (44.3)
Overweight, 25.0–29.9	71,352 (34.4)
Obese, ≥30	39,886 (19.3)
**Age at menarche**	
≤11 years	35,434 (18.1)
12 years	37,801 (19.3)
13 years	49,466 (25.2)
14 years	40,329 (20.6)
≥15 years	33,017 (16.8)
**Number of children**	
0	33,125 (16.2)
1	25,815 (12.6)
2	87,659 (42.9)
≥3	57,922 (28.3)
**Age at natural menopause, mean ± SD**	50.2 ± 4.4
**Age at natural menopause**	
<40 years	3,895 (1.9)
40–44 years	15,134 (7.3)
45–49 years	51,706 (25.0)
50–51 years	50,736 (24.5)
≥52 years	85,760 (41.4)
**Smoking status**	
Never smoker	119,072 (57.5)
Former smoker	63,715 (30.7)
Current smoker	24,444 (11.8)
Intensity of smoking (cigarettes/day) (*n* = 58,843)*	
Former smokers, mean ± SD	16.0 ± 9.1
Current smokers, mean ± SD	14.6 ± 8.4
Duration of smoking (years) (*n* = 59,290)*	
Former smokers, mean ± SD	19.4 ± 10.1
Current smokers, mean ± SD	29.6 ± 7.0
Cumulative dose of smoking (pack-years) (*n* = 57,772)*	
Former smokers, mean ± SD	16.7 ± 13.0
Current smokers, mean ± SD	22.9 ± 13.7
Age started smoking (years) (*n* = 63,695)*	
Former smokers, mean ± SD	18.4 ± 4.4
Current smokers, mean ± SD	19.5 ± 6.6
Years since quitting smoking (*n* = 39,823),* **mean ± SD**	16.3 ± 9.1

*The numbers were based on the available number of women who reported that information.

### Cross-sectional associations

**Fig 1:**
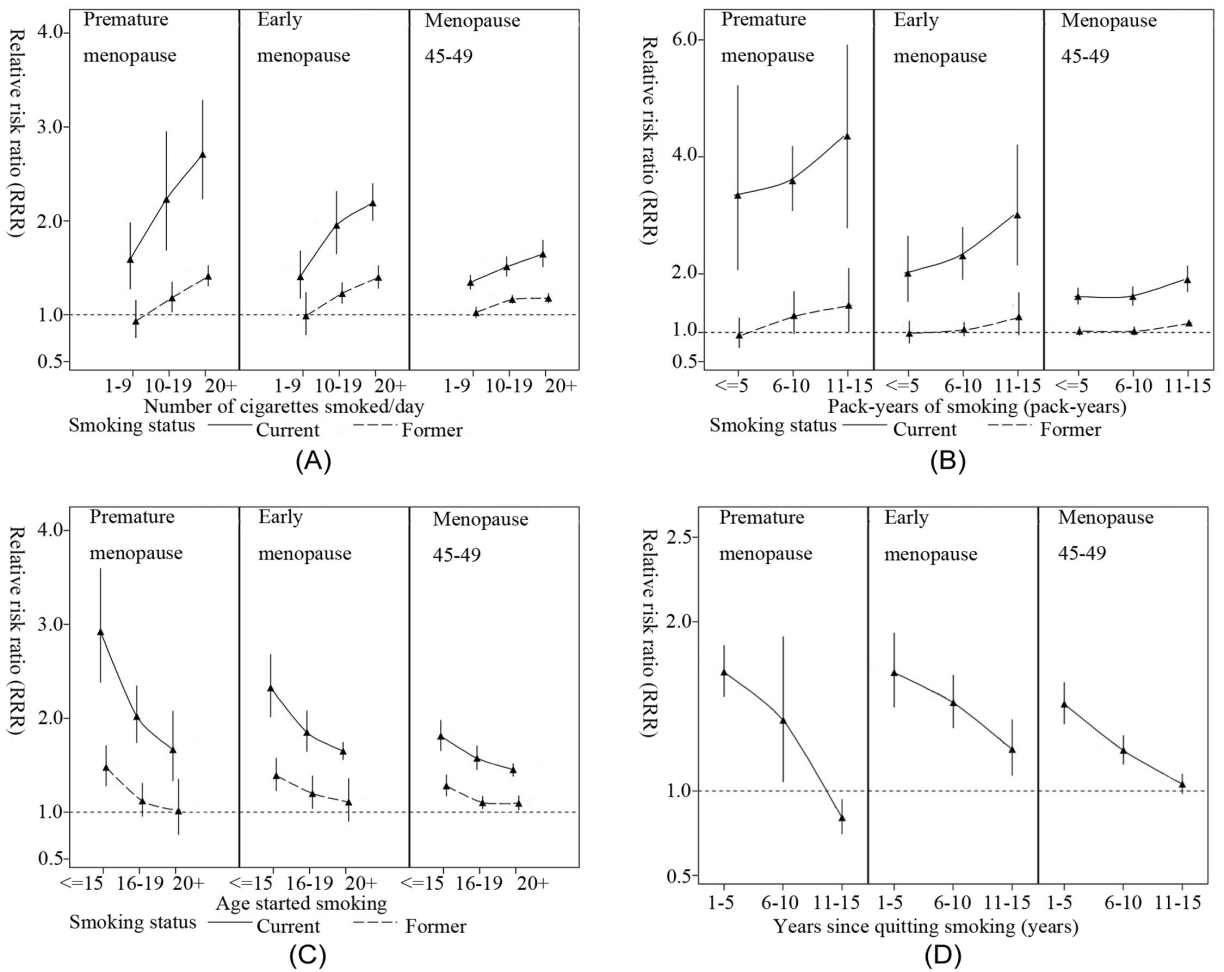
Relationship between smoking exposure and age at natural menopause. The cross-sectional associations of (A) intensity of smoking (cigarettes/day), (B) cumulative dose of smoking (pack-years), (C) age started smoking (years), and (D) years since quitting smoking (years) with the risk for premature menopause (<40 years), early menopause (40–44 years), and menopause at 45–49 years in current and/or former smokers. All RRRs were adjusted for race/ethnicity/region, education level, and BMI. Never smokers were used as reference group. RRR, relative risk ratio.

Compared with never smokers, current smokers experienced an increased risk of premature (RRR 2.05, 95% CI 1.73–2.44; *p* < 0.001) and early (1.80; 1.66–1.95; *p* < 0.001) menopause as well as earlier menopause at 45–49 years (1.47; 1.42–1.52; *p* < 0.001). In former smokers, the corresponding RRRs were substantially attenuated to 1.13 (1.04–1.23; *p* = 0.006), 1.15 (1.05–1.27; *p* = 0.005), and 1.09 (1.05–1.13; *p* < 0.001), respectively (Table 3). A consistent trend was found between all measures of smoking characteristics and age at menopause. Current smokers with long duration of smoking had much higher risk of premature menopause and early menopause, with RRR of 15.58 (95% CI 11.29–19.86; *p* < 0.001) and 6.55 (95% CI 5.04–8.52; *p* < 0.001), respectively, in duration 15–20 years. In both current and former smokers, dose-response relationships were observed in all measures of smoking characteristics, i.e., higher intensity, longer duration, higher cumulative dose, earlier age at start smoking, and shorter time since quitting smoking were significantly associated with higher risk of premature and early menopause, as well as earlier menopause at 45–49 years (Table 3, Fig 1). Compared with never smokers, women who had recently quit smoking (within 5 years) had an increased risk of premature (1.70; 1.56–1.86; *p* < 0.001) and early (1.70; 1.49–1.93; *p* < 0.001) menopause, which were close to the levels of risk seen in current smokers (Table 3). Duration of smoking had the lowest BIC value (366,427), followed by cumulative dose (381,329). When further adjusted for age at menarche and parity, the estimates for smoking status were attenuated slightly (S1 Table).

**Table 3:** Cross-sectional associations between cigarette smoking and age at menopause (*n* = 207,231).

	*N*	Age in years at menopause, *n* (%)					Adjusted RRR (95% CI)*			
		<40	40–44	45–49	50–51	≥52	<40	40–44	45–49	≥52
**Smoking status**										
Never smoker	119,072	1,965 (1.7)	7,776 (6.5)	27,950 (23.5)	29,674 (24.9)	51,707 (43.4)	1.00	1.00	1.00	1.00
Former smoker	63,715	1,161 (1.8)	4,705 (7.4)	15,656 (24.6)	15,391 (24.2)	26,802 (42.1)	1.13 (1.04–1.23)	1.15 (1.05–1.27)	1.09 (1.05–1.13)	0.98 (0.95–1.00)
Current smoker	24,444	769 (3.1)	2,653 (10.9)	8,100 (33.1)	5,671 (23.2)	7,251 (29.7)	2.05 (1.73–2.44)	1.80 (1.66–1.95)	1.47 (1.42–1.52)	0.75 (0.72–0.79)
**Intensity of smoking, cigarettes/day**										
Never smoker	119,072	1,965 (1.7)	7,776 (6.5)	27,950 (23.5)	29,674 (24.9)	51,707 (43.4)	1.00	1.00	1.00	1.00
Former smokers + 1–9 cigs/day	7,933	116 (1.5)	495 (6.2)	1,932 (24.4)	1,957 (24.7)	3,433 (43.3)	0.93 (0.75–1.16)	0.99 (0.78–1.24)	1.02 (0.96–1.08)	1.02 (0.97–1.07)
Former smokers + 10–19 cigs/day	14,892	276 (1.9)	1,148 (7.7)	3,796 (25.5)	3,525 (23.7)	6,147 (41.3)	1.18 (1.03–1.35)	1.23 (1.12–1.35)	1.16 (1.12–1.21)	0.97 (0.91–1.04)
Former smokers + 20 or more cigs/day	16,884	392 (2.3)	1,522 (9.0)	4,342 (25.7)	4,013 (23.8)	6,615 (39.2)	1.41 (1.30–1.53)	1.40 (1.28–1.53)	1.17 (1.12–1.23)	0.90 (0.87–0.93)
Current smokers + 1–9 cigs/day	4,594	113 (2.5)	399 (8.7)	1,435 (31.2)	1,092 (23.8)	1,555 (33.8)	1.59 (1.27–1.98)	1.40 (1.17–1.68)	1.34 (1.27–1.42)	0.84 (0.80–0.89)
Current smokers + 10–19 cigs/day	8,335	285 (3.4)	989 (11.9)	2,823 (33.9)	1,918 (23.0)	2,320 (27.8)	2.23 (1.68–2.95)	1.95 (1.65–2.32)	1.51 (1.41–1.62)	0.71 (0.67–0.76)
Current smokers + 20 or more cigs/day	6,205	255 (4.1)	799 (12.9)	2,173 (35.0)	1,367 (22.0)	1,611 (26.0)	2.71 (2.23–3.29)	2.19 (2.00–2.40)	1.64 (1.51–1.80)	0.68 (0.63–0.74)
**Duration of smoking before menopause, years**										
Never smoker	119,072	1,965 (1.7)	7,776 (6.5)	27,950 (23.5)	29,674 (24.9)	51,707 (43.4)	1.00	1.00	1.00	1.00
Former smokers + duration <10	9,064	150 (1.7)	551 (6.1)	2,175 (24.0)	2,257 (24.9)	3,931 (43.4)	1.00 (0.77–1.29)	0.94 (0.88–1.01)	0.99 (0.91–1.08)	1.03 (0.97–1.08)
Former smokers + duration 10–14	6,551	149 (2.3)	453 (6.9)	1,551 (23.7)	1,637 (25.0)	2,761 (42.1)	1.35 (0.95–1.92)	1.05 (0.88–1.26)	1.00 (0.95–1.05)	0.96 (0.93–1.00)
Former smokers + duration 15–20	5,798	199 (3.4)	506 (8.7)	1,394 (24.0)	1,337 (23.1)	2,362 (40.7)	2.25 (1.50–3.37)	1.44 (1.18–1.75)	1.12 (1.05–1.18)	0.99 (0.93–1.05)
Current smokers + duration <10	295	27 (9.2)	42 (14.2)	114 (38.6)	48 (16.3)	64 (21.7)	9.22 (5.56–15.28)	3.48 (2.04–5.94)	2.26 (2.06–2.47)	0.76 (0.37–1.59)
Current smokers + duration 10–14	596	109 (18.3)	115 (19.3)	177 (29.7)	100 (16.8)	95 (15.9)	14.34 (10.37–18.49)	4.53 (2.91–7.05)	1.90 (1.58–2.28)	0.55 (0.46–0.65)
Current smokers + duration 15–20	1,138	223 (19.6)	276 (24.3)	359 (31.5)	170 (14.9)	110 (9.7)	15.58 (11.29–19.86)	6.55 (5.04–8.52)	2.58 (1.98–3.36)	0.38 (0.28–0.50)
**Cumulative dose of smoking, pack-years**										
Never smoker	119,072	1,965 (1.7)	7,776 (6.5)	27,950 (23.5)	29,674 (24.9)	51,707 (43.4)	1.00	1.00	1.00	1.00
Former smokers + pack-years ≤5	7,310	104 (1.4)	443 (6.1)	1,697 (23.2)	1,794 (24.5)	3,272 (44.8)	0.96 (0.73–1.24)	0.98 (0.81–1.19)	1.02 (0.95–1.10)	1.04 (0.97–1.11)
Former smokers + pack-years 6–10	6,288	128 (2.0)	420 (6.7)	1,456 (23.2)	1,534 (24.4)	2,750 (43.7)	1.29 (0.97–1.70)	1.05 (0.93–1.17)	1.02 (0.94–1.10)	1.00 (0.97–1.04)
Former smokers + pack-years 11–15	7,603	176 (2.3)	603 (7.9)	1,960 (25.8)	1,819 (23.9)	3,045 (40.0)	1.46 (1.01–2.10)	1.27 (1.06–1.71)	1.16 (1.12–1.20)	0.94 (0.91–0.97)
Current smokers + pack-years <5	1,213	57 (4.7)	140 (11.5)	414 (34.1)	274 (22.6)	328 (27.0)	3.34 (2.07–5.22)	2.01 (1.52–2.65)	1.61 (1.48–1.76)	0.69 (0.59–0.80)
Current smokers + pack-years 6–10	2,135	103 (4.8)	268 (12.6)	682 (31.9)	452 (21.2)	630 (29.5)	3.59 (3.08–4.19)	2.31 (1.90–2.80)	1.61 (1.45–1.78)	0.81 (0.68–0.95)
Current smokers + pack-years 11–15	2,765	157 (5.7)	440 (15.9)	1,008 (36.5)	570 (20.6)	590 (21.3)	4.35 (2.78–5.92)	3.01 (2.15–4.21)	1.90 (1.69–2.14)	0.60 (0.51–0.70)
**Age started smoking, years**										
Never smoker	119,072	1,965 (1.7)	7,776 (6.5)	27,950 (23.5)	29,674 (24.9)	51,707 (43.4)	1.00	1.00	1.00	1.00
Former smokers + age started at ≥20	11,593	184 (1.6)	812 (7.0)	2,958 (25.5)	2,883 (24.9)	4,756 (41.0)	1.01 (0.76–1.35)	1.10 (0.90–1.36)	1.09 (1.02–1.18)	0.94 (0.86–1.03)
Former smokers + age started at 16–19	22,814	407 (1.8)	1,738 (7.6)	5,608 (24.6)	5,478 (24.0)	9,583 (42.0)	1.12 (0.95–1.31)	1.20 (1.03–1.39)	1.10 (1.03–1.17)	0.98 (0.95–1.00)
Former smokers + age started at ≤15	8,740	215 (2.5)	781 (8.9)	2,426 (27.8)	2,035 (23.3)	3,283 (37.6)	1.48 (1.27–1.71)	1.39 (1.22–1.57)	1.28 (1.17–1.40)	0.89 (0.86–0.93)
Current smokers + age started at ≥20	7,484	187 (2.5)	753 (10.1)	2,531 (33.8)	1,846 (24.7)	2,167 (29.0)	1.66 (1.33–2.08)	1.65 (1.56–1.75)	1.45 (1.38–1.53)	0.70 (0.62–0.79)
Current smokers + age started at 16–19	8,399	259 (3.1)	936 (11.1)	2,831 (33.7)	1,934 (23.0)	2,439 (29.0)	2.02 (1.74–2.35)	1.85 (1.64–2.08)	1.57 (1.45–1.71)	0.72 (0.69–0.76)
Current smokers + age started at ≤15	4,665	210 (4.5)	616 (13.2)	1,644 (35.2)	974 (20.9)	1,221 (26.2)	2.92 (2.38–3.60)	2.32 (2.01–2.68)	1.81 (1.65–1.98)	0.72 (0.66–0.77)
**Years since quitting smoking before menopause, years**										
Never smoker	119,072	1,965 (1.7)	7,776 (6.5)	27,950 (23.5)	29,674 (24.9)	51,707 (43.4)	1.00	1.00	1.00	1.00
Current smoker	24,444	769 (3.1)	2,653 (10.9)	8,100 (33.1)	5,671 (23.2)	7,251 (29.7)	2.10 (1.79–2.45)	1.82 (1.73–1.92)	1.53 (1.46–1.61)	0.73 (0.67–0.78)
1–5	7,279	218 (3.0)	792 (10.9)	2,303 (31.6)	1,729 (23.8)	2,237 (30.7)	1.70 (1.56–1.86)	1.70 (1.49–1.93)	1.51 (1.39–1.64)	0.82 (0.77–0.87)
6–10	7,107	177 (2.5)	637 (8.9)	1,947 (27.4)	1,585 (22.3)	2,761 (38.9)	1.42 (1.05–1.91)	1.52 (1.37–1.69)	1.24 (1.16–1.33)	0.87 (0.80–0.94)
11–15	7,752	140 (1.8)	668 (8.6)	1,944 (25.3)	2,037 (26.2)	2,963 (38.1)	0.84 (0.74–0.95)	1.25 (1.09–1.42)	1.04 (0.98–1.10)	0.81 (0.78–0.85)

*Multinomial logistic regression model was used to estimate RRR and 95% CIs with the category of 50–51 years as reference. All RRRs were adjusted for race/ethnicity/region, education level, and BMI.

**Abbreviations:** CI, confidence interval; cigs, cigarettes; RRR, relative risk ratio.

### Sensitivity analyses and meta-analyses

After excluding the UK Biobank data, the associations in former smokers with premature (1.04, 0.86–1.26; *p* = 0.641) and early (1.01; 0.87–1.18; *p* = 0.730) menopause were no longer significant (S2 Table). In addition, when intensity, duration, and age at start of smoking were analysed by further adjusting for other smoking characteristics (S3 Table), a similar set of relationships were found as when the UK Biobank data were included, except that for current smokers the risk of early menopause was no longer significant (1.28; 0.91–1.81; *p* = 0.061) compared with the reference group (women who had quit smoking for at least 11 years). The study-specific regressions and random-effects meta-analysis for the associations between each level of cigarettes smoking exposures and each category of menopause showed consistent results with the main analysis (S4 Table).

### Prospective associations

Overall, a similar pattern of results were obtained from prospective analyses that only included women who experienced menopause after baseline. Due to the limited number of women experiencing early menopause (<45 years) at a subsequent survey, some estimates of association for early menopause were attenuated (e.g., cumulative dose of smoking in current smokers) or with wide CIs (e.g., intensity of smoking in former smokers) (Table 4). The study-specific regressions and random-effects meta-analysis for prospective analyses were provided in S5 Table.

**Table 4:** Prospective associations between cigarette smoking and age at menopause (*n* = 27,580).

	*N*	Age in years at menopause, *n* (%)				Adjusted RRR (95% CI)*		
		<45	45–49	50–51	≥52	<45	45–49	≥52
**Smoking status**								
Never smoker	14,401	209 (1.5)	2,535 (17.6)	3,695 (25.7)	7,962 (55.3)	1.00	1.00	1.00
Former smoker	8,337	163 (2.0)	1,714 (20.6)	2,170 (26.0)	4,290 (51.5)	1.21 (1.03–1.41)	1.11 (0.94–1.32)	0.94 (0.85–1.03)
Current smoker	4,842	141 (2.9)	1,425 (29.4)	1,257 (26.0)	2,019 (41.7)	1.67 (1.32–2.11)	1.55 (1.45–1.66)	0.78 (0.70–0.86)
**Intensity of smoking, cigarettes/day**								
Never smoker	14,401	209 (1.5)	2,535 (17.6)	3,695 (25.7)	7,962 (55.3)	1.00	1.00	1.00
Former smokers + 1–9 cigs/day	1,819	27 (1.5)	335 (18.4)	487 (26.8)	970 (53.3)	1.01 (0.77–1.32)	1.01 (0.72–1.43)	0.93 (0.85–1.02)
Former smokers + 10–19 cigs/day	1,577	30 (1.9)	273 (17.3)	452 (28.7)	822 (52.1)	1.41 (0.74–2.71)	0.95 (0.69–1.31)	0.82 (0.69–0.96)
Former smokers + 20 or more cigs/day	1,445	40 (2.8)	323 (22.4)	384 (26.6)	698 (48.3)	1.99 (0.98–4.03)	1.24 (0.88–1.74)	0.85 (0.62–1.16)
Current smokers + 1–9 cigs/day	864	18 (2.1)	220 (25.5)	241 (27.9)	385 (44.6)	1.25 (0.76–2.05)	1.28 (1.05–1.57)	0.76 (0.69–0.85)
Current smokers + 10–19 cigs/day	1,553	56 (3.6)	485 (31.2)	397 (25.6)	615 (39.6)	2.11 (1.66–2.68)	1.68 (1.54–1.82)	0.74 (0.62–0.88)
Current smokers + 20 or more cigs/day	1,475	43 (2.9)	470 (31.9)	377 (25.6)	585 (39.7)	2.14 (1.60–2.88)	1.90 (1.49–2.43)	0.72 (0.65–0.80)
**Duration of smoking before menopause,**^†^ **years**								
Never smoker	14,401	209 (1.5)	2,535 (17.6)	3,695 (25.7)	7,962 (55.3)	1.00	1.00	1.00
Former smokers + duration <10	2,635	48 (1.8)	525 (19.9)	700 (26.6)	1,362 (51.7)	1.12 (0.68–1.84)	1.06 (0.91–1.23)	0.91 (0.82–1.02)
Former smokers + duration 10–20	1,337	25 (1.9)	302 (22.6)	348 (26.0)	662 (49.5)	1.17 (0.89–1.54)	1.15 (1.01–1.31)	0.89 (0.79–1.02)
Current smokers + duration <10	79	10 (12.7)	30 (38.0)	17 (21.5)	22 (27.8)	11.53 (6.01–22.18)	2.74 (1.83–4.12)	0.60 (0.40–0.90)
Current smokers + duration 10–20	260	31 (11.9)	112 (43.1)	48 (18.5)	69 (26.5)	11.54 (7.00–19.01)	3.36 (2.29–4.93)	0.68 (0.45–1.01)
**Cumulative dose of smoking,**^‡^ **pack-years**								
Never smoker	14,401	209 (1.5)	2,535 (17.6)	3,695 (25.7)	7,962 (55.3)	1.00	1.00	1.00
Former smokers + pack-years ≤10	1,781	31 (1.7)	288 (16.2)	478 (26.8)	984 (55.2)	1.15 (0.90–1.47)	0.88 (0.69–1.12)	0.97 (0.87–1.08)
Former smokers + pack-years 11–15	2,026	45 (2.2)	490 (24.2)	543 (26.8)	948 (46.8)	1.28 (1.03–1.60)	1.16 (0.93–1.45)	0.83 (0.76–0.90)
Current smokers + pack-years ≤10	441	10 (2.3)	118 (26.8)	120 (27.2)	193 (43.8)	1.69 (1.00–2.88)	1.45 (0.97–2.18)	0.77 (0.65–0.90)
Current smokers + pack-years 11–15	771	27 (3.5)	234 (30.4)	193 (25.0)	317 (41.1)	2.29 (1.45–3.61)	1.70 (1.39–2.08)	0.77 (0.61–0.98)
**Age started smoking, years**								
Never smoker	14,401	209 (1.5)	2,535 (17.6)	3,695 (25.7)	7,962 (55.3)	1.00	1.00	1.00
Former smokers + age started at ≥20	1,458	14 (1.0)	261 (17.9)	392 (26.9)	791 (54.3)	0.62 (0.38–1.03)	0.96 (0.78–1.17)	0.94 (0.83–1.07)
Former smokers + age started at 16–19	3,618	48 (1.3)	686 (19.0)	992 (27.4)	1,892 (52.3)	0.86 (0.66–1.11)	1.00 (0.81–1.24)	0.88 (0.80–0.97)
Former smokers + age started at ≤15	1,863	79 (4.2)	488 (26.2)	465 (25.0)	831 (44.6)	2.65 (2.00–3.52)	1.46 (1.03–2.07)	0.83 (0.76–0.92)
Current smokers + age started at ≥20	1,074	19 (1.8)	277 (25.8)	285 (26.5)	493 (45.9)	1.12 (0.55–2.28)	1.38 (1.15–1.66)	0.82 (0.70–0.95)
Current smokers + age started at 16–19	1,945	52 (2.7)	541 (27.8)	546 (28.1)	806 (41.4)	1.57 (1.17–2.09)	1.41 (1.28–1.56)	0.69 (0.63–0.77)
Current smokers + age started at ≤15	1,316	59 (4.5)	463 (35.2)	318 (24.2)	476 (36.2)	2.84 (2.35–3.42)	2.03 (1.85–2.24)	0.71 (0.57–0.88)
**Years since quitting smoking before menopause**^§^								
Never smoker	14,401	209 (1.5)	2,535 (17.6)	3,695 (25.7)	7,962 (55.3)	1.00	1.00	1.00
Current smoker	4,210	133 (3.2)	1,275 (30.3)	1,109 (26.3)	1,693 (40.2)	1.97 (1.56–2.50)	1.63 (1.49–1.79)	0.72 (0.65–0.80)
1–5	692	20 (2.9)	190 (27.5)	166 (24.0)	316 (45.7)	1.70 (0.92–3.15)	1.52 (1.19–1.93)	0.90 (0.73–1.11)
6–15	1,179	33 (2.5)	318 (26.5)	268 (24.8)	560 (46.2)	1.52 (1.01–2.29)	1.40 (1.17–1.67)	0.88 (0.76–1.02)
15+	4,425	33 (0.8)	678 (15.1)	1,202 (26.7)	2,512 (57.5)	0.86 (0.51–1.47)	1.00 (0.85–1.19)	0.74 (0.62–0.88)

*Multinomial logistic regression model was used to estimate RRR and 95% Cis, with the category of 50–51 years as reference. All RRRs were adjusted for race/ethnicity/region, education level, and BMI.

^†^The categories of 10–14 years and 15–20 years were combined as 10–20 years for analysis for limited number of women with early menopause.

^‡^The categories of ≤5 pack-years and 6–10 pack-years were combined as ≤10 pack-years for analysis for limited number of women with early menopause.

^§^The categories of 6–10 and 11–15 were combined as 6–15 for analysis due to the insufficient number of women with early menopause.

**Abbreviations:** CI, confidence interval; cigs, cigarettes; RRR, relative risk ratio.

## Discussion

### Summary of results

Our results indicate that compared with never smokers, both current smokers and former smokers are at higher risk of premature and early menopause and menopause at 45–49 years, although the association is weaker among former smokers. Intensity, duration, cumulative dose, younger age at start smoking, and shorter time since quitting smoking are all associated with earlier age at menopause in both current and former smokers, but the excess risks were substantially lower in former smokers. Consistent dose-response relationships were observed in both current and former smokers. Smoking duration is a much stronger predictor of earlier menopause than others. Current smokers with long duration (15–20 years) showed particularly higher risk of premature and early menopause. These findings highlight the clear benefits of early smoking cessation to lower the excess risk of earlier menopause.

### Former smokers and age at menopause

Current smoking is an established risk factor for earlier menopause [3,15,16], whereas the relationship between past smoking and age at menopause is less conclusive. Compared with never smokers, some previous studies showed that former smokers had earlier menopause [20,25], others found no association [26–28], and one study a reduced risk [29]. The number of former smokers in studies that found no association was small (around 1,000 women) [26,28], and the study that found a negative association had only 83 former smokers [29]. We found that being a former smoker was significantly associated with premature and early menopause, as well as menopause at 45–49 years, indicating that the effect of smoking on ovarian senescence is not limited to current smoking.

### Potential mechanisms linking smoking to earlier menopause

Several hypotheses might explain why smoking leads to earlier menopause. First, cigarette smoke yields polycyclic aromatic hydrocarbons (PAHs), which activate the aromatic hydrocarbon receptor. This receptor induces expression of the apoptosis-promoting gene Bcl2-associated X protein (BAX) in oocytes, which increases the rate of oocyte apoptosis leading to earlier ovarian failure [30]. This mechanism indicates a permanent, irreversible influence that occurs in both former and current smokers and may explain why former smokers have an earlier menopause than never smokers. PAHs also induce other hepatic enzymes, such as monooxygenases and transferases, which enhance steroid metabolism and clearance [31]. Furthermore, PAHs induce or up-regulate the expression of cytochrome P450 enzymes to solubilize and decrease the serum estrogen levels [32]. Second, smoking has been suggested to have an anti-estrogenic effect [33]; awaking the 2-hydroxylation pathway in the liver would convert bioactive estrogens into 2-hydroxyestrogens with minimal activity in peripheral tissues [34]. Finally, smoking increases adrenal production of androgens, which resists or blunts the functions of estrogens [35]. The latter two mechanisms reflect a temporary and reversible effect and may explain why being a current smoker has a greater impact on earlier menopause than being a former smoker.

### Intensity, duration, quantity, and timing of smoking and age at menopause

Some studies have reported a potential dose-response relationship between intensity of smoking and earlier menopause [17,18,28], while other studies have shown no such association [27,29]. A systematic review including virtually every informative study indicated that the dose-response gradient was slight and nonlinear [16]. However, limited by the significant heterogeneity between studies, this review neither gave overall estimates nor did it separate the estimates for current and former smokers. In contrast, we observed a clear dose-response relationship in both current and former smokers, compatible with the toxic effect of smoking on oocyte quantity. Experimental studies using animal models have shown that exposure to tobacco smoke causes follicle loss at all stages of folliculogenesis [36], and a dose-response trend exists for nicotine on impairment in follicle growth [37].

Evidence of the association between duration of smoking and age at menopause has been inconsistent [17,18,26]. A recent large-scale study reported a possible dose-response relationship between smoking duration and earlier menopause [18]. However, the analyses were performed in all smokers, and thus the specific associations of smoking duration among former or current smokers could not be examined. By definition, smoking duration is somewhat confounded with former/current smoking status, warranting separate analysis. Also, in that study [18], because age at menopause was categorized only into two categories of <50 and ≥50, the association with premature or early menopause could not be examined. We found that prolonged smoking was linked to earlier menopause, both in former and current smokers. The associations of smoking duration with premature and early menopause were much higher in current smokers than that in former smokers. The lowest BIC value indicates that duration of smoking is the strongest predictor of earlier age at menopause in this study.

Number of pack-years reflects a person’s cumulative cigarette exposure over time. Of the four cross-sectional studies that examined the association between pack-years and age at menopause [17,18,30,38], three suggested a slight dose-response relationship [17,18,38], with a higher number of pack-years associated with earlier menopause. However, all three studies used data from all smokers without stratifying by current or former smoking status. Our results showed that higher cumulative dose of smoking was strongly associated with earlier menopause in both current and former smokers.

Some studies found a dose-response relationship with younger age at starting to smoke and earlier age at menopause [17,18], while others did not detect a significant association [20,39]. We found that the earlier age at starting to smoke, the higher the risk of experiencing earlier menopause both among current and former smokers, especially for women who started smoking before age 15. Similar to one previous study [20], we found that the more recently women had quit smoking, the higher the risk of earlier menopause. This finding indicated that the residual influence of smoking on earlier menopause declined as years since quitting smoking increased.

### Limitations

Some limitations need to be considered when interpreting these results. Current smoking status was determined at the baseline survey. For women who experienced menopause after baseline, their smoking status was measured at varying years before menopause. Thus, the possibility of measurement errors in smoking status may have arisen. Smoking, however, is a habit that is resistant to change. In studies including women who reported smoking status both before and after menopause (e.g., UK Biobank, NSHD, NCDS, SWAN, and SABRE), the concordance was over 83%, supporting the use of smoking exposure at baseline as a proxy for smoking status close to or after menopause. For example, when van Asselt and colleagues [26] repeated their analyses by defining current and former smokers at 5 years before menopause instead of 1 year, the estimated effects were similar. This suggests that among women with menopause after baseline in this study (the mean baseline age and menopausal age for these women were 46.0 and 51.5 years, respectively), our estimates reflect the effect of smoking exposure just before or close to menopausal age. Another limitation was that the influence of passive smoking, which may lead to earlier menopause [18], was not considered in this study; thus, when the never-smoking group was used as the reference without excluding passive smokers, the association might have been underestimated. However, SWAN study found that both baseline and time-varying passive smoking did not predict age at menopause [3], so we assume that the bias from not including passive smoking is small. Third, age at menopause was self-reported. However, because premature and early menopause are landmark health events, we consider major misclassification unlikely. Fourth, the original questions used to collect smoking data may not be exactly the same across studies, which may lead to some heterogeneity in the responses. Based on validation studies, smoking questions that have a reasonably standard format (e.g., questions about smoking status or duration of smoking) are not expected to impact the findings in any substantive way [40,41]. Finally, over 80% women are white in our study, which may limit the generalizability of the findings to other races.

### Strengths of this study

The main strength of this study was the use of pooled individual-level data from 17 studies across different geographic regions and populations, which resulted in sufficient statistical power to perform both cross-sectional and prospective analyses in current and former smokers separately. The percentages of women with missing data on smoking status, BMI, education level, and race/ethnicity were relatively small (ranging from 1.3% to 5.4%). To our knowledge, it is the first study of this size to examine and quantify the dose-response relationships between intensity, duration, cumulative dose, timing of smoking, and age at menopause separately for former and current smokers. Also, the participant-level data in InterLACE enabled harmonizing variables using common definitions, coding and cut points, which is not usually possible with meta-analyses of published results.

### Conclusion

Our pooled analysis provides robust evidence that both former and current smokers are at an increased risk of experiencing earlier menopause, but the risk is considerably lower for former smokers. We found significant relationships between the intensity, duration, cumulative dose, the timing of starting, and years since quitting smoking with the risk of earlier menopause in both current and former smokers. Duration and cumulative dose of smoking are good predictors of age at menopause. Current smokers with 15 or more years of smoking duration experience a 15-fold increased risk of premature and a 6-fold increased risk of early menopause. Given that 175 million women ages 15 or older are current smokers globally [42], our findings highlight the clear benefits for women of early smoking cessation to lower their excess risk of earlier menopause, thereby reducing the increased risks of chronic diseases (e.g., cardiovascular disease and osteoporosis) associated with both smoking and earlier menopause. Furthermore, smoking cessation itself reduces the risk of many other smoking-related diseases, such as lung cancer and chronic obstructive pulmonary disease. In our future research, we intend to quantify the direct and indirect effects (via age at menopause) of smoking on the risk of chronic diseases.

## Supporting information

S1 Text STROBE Statement—checklist of items that should be included in reports of observational studies. (DOC)

S2 Text Prospective research proposal. (DOCX)

S3 Text Funding details of studies contributed to the InterLACE consortium. (DOCX)

S1 Table Cross-sectional associations between cigarette smoking and age at menopause, further adjusted age at menarche and parity in 14 studies (*n* = 194,368). (DOCX)

S2 Table Cross-sectional associations between cigarette smoking and age at menopause after excluding UK Biobank study (*n* = 69,217). (DOCX)

S3 Table Cross-sectional association between intensity, duration, age at start of smoking, and age at menopause-adjusted other smoking factors. (DOCX)

S4 Table Meta-analysis results from study-specific cross-sectional associations between cigarette smoking and age at menopause. (DOCX)

S5 Table Meta-analysis results from study-specific prospective associations between cigarette smoking and age at menopause. (DOCX)

S6 Table List of contacts for data access. (DOCX)

## Abbreviations

ALSWH: Australian Longitudinal Study on Women’s Health
BAX: Bcl2-associated X protein
BIC: Bayesian information criterion
BMI: body mass index
CI: confidence interva
DNC: Danish Nurse Cohort Study
ELSA: English Longitudinal Study of Ageing
French 3C: French Three-City Study
HILO: Hilo Women’s Healthy Study
HOW: Healthy Ageing of Women Study
HT: hormone therapy
InterLACE: International collaboration for a Life course Approach to reproductive health and Chronic disease Events
JNHS: Japan Nurses’ Health Study
MCCS: Melbourne Collaborative Cohort Study
NCDS: National Child Development Study
NSHD: National Survey of Health and Development
OCP: oral contraceptive pill
PAH: polycyclic aromatic hydrocarbon
POF: premature ovarian failure
RRR: relative risk ratio
SABRE: Southall And Brent Revisited
SMWHS: Seattle Middle Women’s Health Study
STROBE: Strengthening the Reporting of Observational Studies in Epidemiology
SWAN: Study of Women's Health Across the Nation
UKWCS: UK Women’s Cohort Study
WHITEHALL: Whitehall II study
WHO: World Health Organization
WLH: Women’s Lifestyle and Health Study
