# Supplementary material for: Relationships between intensity, duration, cumulative dose, and timing of smoking with age at menopause: A pooled analysis of individual data from 17 observational studies
Source: PLoS Med. 2018 Nov 27;15(11):e1002704. doi: 10.1371/journal.pmed.1002704 (PMC6258514; doi:10.1371/journal.pmed.1002704)
Supplement: S1 Text — (DOC) [file pmed.1002704.s001.doc]

**S1 Text. STROBE Statement—checklist of items that should be included in reports of observational studies**

|  | | Item No | Recommendation |  | Relevant content in this paper |
| --- | --- | --- | --- | --- | --- |
| **Title and abstract** | | 1 | (*a*) Indicate the study’s design with a commonly used term in the title or the abstract |  | In Title: ‘a pooled analysis in 17 observational studies’.  In Abstract: ‘the cross-sectional and prospective analyses’ in Methods and findings section. |
|  |  |  | (*b*) Provide in the abstract an informative and balanced summary of what was done and what was found |  | In the Methods and findings section of Abstract. |
| Introduction | | | |  |  |
| Background/rationale | | 2 | Explain the scientific background and rationale for the investigation being reported |  | Paragraph 1 and 2 in Introduction |
| Objectives | | 3 | State specific objectives, including any prespecified hypotheses |  | Paragraph 3 in Introduction |
| Methods | | | |  |  |
| Study design | | 4 | Present key elements of study design early in the paper |  | Paragraph 1 in Methods: ‘InterLACE has pooled individual-level data on reproductive health and chronic diseases from 25 observational studies across ten countries. Most studies are prospective’. |
| Setting | | 5 | Describe the setting, locations, and relevant dates, including periods of recruitment, exposure, follow-up, and data collection |  | Paragraph 2 in Methods: ‘17 studies of InterLACE (in ten countries Table 1) provided information on women’s smoking status at the baseline survey and age at natural menopause’. |
| Participants | | 6 | (*a*) *Cohort study*—Give the eligibility criteria, and the sources and methods of selection of participants. Describe methods of follow-up  *Case-control study*—Give the eligibility criteria, and the sources and methods of case ascertainment and control selection. Give the rationale for the choice of cases and controls  *Cross-sectional study*—Give the eligibility criteria, and the sources and methods of selection of participants |  | Paragraph 2 in Methods: ‘The cross-sectional analysis was based on 207 231 postmenopausal women with information on age at natural menopause, smoking status, and key covariates at baseline, including body mass index (BMI), years of education, and race/ethnicity/region. The prospective analysis was based on 27 580 women who experienced menopause after the baseline survey.’ |
|  |  |  | (*b*) *Cohort study*—For matched studies, give matching criteria and number of exposed and unexposed  *Case-control study*—For matched studies, give matching criteria and the number of controls per case |  | \| Not applicable (the current study is not a matched study). \| \| --- \| |
| Variables | | 7 | Clearly define all outcomes, exposures, predictors, potential confounders, and effect modifiers. Give diagnostic criteria, if applicable |  | These are defined and presented in the ‘**Outcome and exposure variables**’ and ‘**Covariates**’ sections in Methods part. **The outcome** was categorised age at menopause: <40 years (premature menopause), 40-44 (early menopause), 45-49, 50-51 (reference category) and 52 years and above.  **The exposures** included multiple aspects of smoking measures: smoking status (current/former/never), cigarettes smoked per day (intensity), smoking duration, pack-years (cumulative dose), age started and years since quitting smoking.  **The covariates** included baseline body mass index (BMI), years of education, race/ethnicity, parity, and age at menarche. |
| Data sources/ measurement | | 8* | For each variable of interest, give sources of data and details of methods of assessment (measurement). Describe comparability of assessment methods if there is more than one group |  | These are presented in the ‘**Outcome and exposure variables**’ section of Methods part. Age at menopause was self-reported and excluded hormone therapy (HT) users and oral contraceptive pills (OCPs) users before menopause.  Smoking status, intensity, duration, age started and years since quitting of smoking were self-reported at baseline. Pack-years of smoking was calculated by using intensity and duration of smoking. |
| Bias | | 9 | Describe any efforts to address potential sources of bias |  | These are presented in the ‘**Sensitivity analyses and two-step meta-analyses**’ section of Methods part.  To address potential sources of bias, we firstly conducted a sensitivity analysis by excluding UK Biobank study whose data contributed more than 60% of the total sample; We also analysed the effect of exposures by adjusting each other; We finally performed two-steps meta-analyses to evaluate the heterogeneity among studies we included.  Besides, to deal with possible measurement errors of smoking exposure caused by recall bias, in studies including women who reported smoking exposure both before and after menopause, we analysed the concordance rate and it was over 83%. |
| Study size | | 10 | Explain how the study size was arrived at |  | Done (Paragraph 2 in Method: Participants section ) |
| Quantitative variables | | 11 | Explain how quantitative variables were handled in the analyses. If applicable, describe which groupings were chosen and why |  | These are presented in the ‘**Outcome and exposure variables**’ and ‘**Covariates**’ sections of Methods part. Age at menopause was categorised as <40 years (premature menopause), 40-44 (early menopause), 45-49, 50-51 (reference category) and 52 years and above.  Smoking intensity was categorised as 1-9, 10-19, and 20 cigarettes or more per day; Duration of smoking was categorised as <10, 10-14, and 15-20 years; Cumulative dose of smoking was categorised as ≤5, 6-10, and 11-15; Age started smoking was categorised as <15, 15-19, 20 or more years of age; years since quitting smoking was categorised as 1-5, 6-10 and 11-15 years. By definition of duration in current smokers, women with later menopause would tend to have a longer duration. In order to observe the effect of smoking duration on earlier menopause, we focused on short duration of smoking. That’s why we chose the present categories for duration (similar reason for categories of pack-years and years since quitting smoking).  We also treated age at menopause and smoking measures as **continuous variables** to examine possible non-linear relationship between them. |
| Statistical methods | | 12 | (*a*) Describe all statistical methods, including those used to control for confounding |  | In the ‘Statistical analyses’ section of Methods part. |
|  |  |  | (*b*) Describe any methods used to examine subgroups and interactions |  | In the ‘Statistical analyses’ section of Methods part. |
|  |  |  | (*c*) Explain how missing data were addressed |  | Analyses were based on complete cases. |
|  |  |  | (*d*) *Cohort study*—If applicable, explain how loss to follow-up was addressed  *Case-control study*—If applicable, explain how matching of cases and controls was addressed  *Cross-sectional study*—If applicable, describe analytical methods taking account of sampling strategy |  | NA |
|  |  |  | (*e*) Describe any sensitivity analyses |  | In the ‘Sensitivity analyses and two-step meta-analyses’ section of Methods part. |
| Results | | | |  |  |
| Participants | 13* | (a) Report numbers of individuals at each stage of study—eg numbers potentially eligible, examined for eligibility, confirmed eligible, included in the study, completing follow-up, and analysed | |  | In the ‘Participants’ section of Methods part |
|  |  | (b) Give reasons for non-participation at each stage | |  | NA |
|  |  | (c) Consider use of a flow diagram | |  |  |
| Descriptive data | 14* | (a) Give characteristics of study participants (eg demographic, clinical, social) and information on exposures and potential confounders | |  | In ‘Baseline characteristics’ section of Results part. |
|  |  | (b) Indicate number of participants with missing data for each variable of interest | |  | Table 2 |
|  |  | (c) *Cohort study*—Summarise follow-up time (eg, average and total amount) | |  | NA |
| Outcome data | 15* | *Cohort study*—Report numbers of outcome events or summary measures over time | |  |  |
|  |  | *Case-control study—*Report numbers in each exposure category, or summary measures of exposure | |  |  |
|  |  | *Cross-sectional study—*Report numbers of outcome events or summary measures | |  | In ‘Baseline characteristics’ section of Results part and Table 2. |
| Main results | 16 | (*a*) Give unadjusted estimates and, if applicable, confounder-adjusted estimates and their precision (eg, 95% confidence interval). Make clear which confounders were adjusted for and why they were included | |  | In the ‘Cross-sectional associations’ and ‘Prospective associations’ section of Results part. Table 3 and 4. |
|  |  | (*b*) Report category boundaries when continuous variables were categorized | |  | Table 3 and 4 |
|  |  | (*c*) If relevant, consider translating estimates of relative risk into absolute risk for a meaningful time period | |  |  |
| Other analyses | 17 | Report other analyses done—eg analyses of subgroups and interactions, and sensitivity analyses | |  | In the ‘Sensitivity analyses and meta-analyses’ section of Results part. |
| Discussion | | | |  |  |
| Key results | 18 | Summarise key results with reference to study objectives | |  | Paragraph 1 of Discussions part: ‘Summary of results’ section. |
| Limitations | 19 | Discuss limitations of the study, taking into account sources of potential bias or imprecision. Discuss both direction and magnitude of any potential bias | |  | In the ‘Limitations’ section of Discussions part. |
| Interpretation | 20 | Give a cautious overall interpretation of results considering objectives, limitations, multiplicity of analyses, results from similar studies, and other relevant evidence | |  | In the ‘Former smokers and age at menopause’ section, ‘Intensity, duration, quantity and timing of smoking and age at menopause’ section and ‘Conclusions’ section. |
| Generalisability | 21 | Discuss the generalisability (external validity) of the study results | |  | In the ‘Limitations’ section: ‘Finally, over 80% women are white in our study, which may limit the generalizability of the findings to other races.’ |
| Other information | | | |  |  |
| Funding | 22 | Give the source of funding and the role of the funders for the present study and, if applicable, for the original study on which the present article is based | |  | In the ‘Funding’ part of the paper. |

*Give information separately for cases and controls in case-control studies and, if applicable, for exposed and unexposed groups in cohort and cross-sectional studies.

**Note:** An Explanation and Elaboration article discusses each checklist item and gives methodological background and published examples of transparent reporting. The STROBE checklist is best used in conjunction with this article (freely available on the Web sites of PLoS Medicine at http://www.plosmedicine.org/, Annals of Internal Medicine at http://www.annals.org/, and Epidemiology at http://www.epidem.com/). Information on the STROBE Initiative is available at www.strobe-statement.org.
