# Supplementary material for: Relationships between intensity, duration, cumulative dose, and timing of smoking with age at menopause: A pooled analysis of individual data from 17 observational studies
Source: PLoS Med. 2018 Nov 27;15(11):e1002704. doi: 10.1371/journal.pmed.1002704 (PMC6258514; doi:10.1371/journal.pmed.1002704)
Supplement: S2 Text — (DOCX) [file pmed.1002704.s002.docx]

**S2 Text. Prospective research proposal**

**Date of commencing the research proposal:** January 09, 2018

**Research title:**

The dose-response relationships between intensity, duration, cumulative dose, and timing of smoking and age at menopause: a pooled analysis of over 200,000 women in 17 observational studies

**Research aim:**

Cigarette smoking is an established risk factor for earlier menopause, but the impact of former smoking on age at natural menopause is inconclusive, and little is known about the nature of the relationship. This study aimed to examine the effect of intensity, duration, quantity, and timing of cigarette smoking on age at menopause in both current and former smokers using data from InterLACE consortium.

**Sample to be used:**

Postmenopausal women who had reported their age at natural menopause, and had reported their smoking status at baseline, and had complete information on the covariates at baseline (Race/ethnicity, Body mass index and Education level) will be included.

**Exclusion criteria:**

1. Women who had missing data on age at menopause;
2. Women who experienced menopause as the result of interventions (such as surgical menopause due to bilateral oophorectomy or hysterectomy);
3. Woman who had missing data on smoking status and key covariates at baseline.

**Dependent/outcome variable:**

- Age at natural menopause: <40 (premature), 40-44 (early), 45-49, 50-51 (reference), and ≥52 years.

**Independent variables:**

- Smoking status: Current smokers, former smokers, never smokers (reference);
- Intensity: Number of cigarettes smoked per day;
- Age started smoking;
- Years since quitting smoking: created by using age at menopause and age stopped smoking;
- Duration of smoking (Created by using age at menopause, age stopped, and age started smoking);
- Pack years: created by using intensity and duration.

**Covariates:**

- Race/ethnicity
- Body mass index
- Education level
- Number of children
- Age at menarche

**Analytic design:**

Cross-sectional analysis and prospective analysis

**Approach to analysis:**

1. Characteristics of women included in the cross-sectional analyses will be presented, including distribution of covariates and smoking measures (smoking status, number of cigarettes smoked per day, duration, age started smoking and pack-years of smoking). Data will be presented as N (%) or mean ± SD.
2. Both cross-sectional and prospective analyses were used. Multinomial logistic regression models with five categories of outcome for age at menopause (<40, 40-44, 45-49, 50-51, 52+) will be used to examine the associations between each categorical form of smoking measures and age at menopause. For age at menopause, category of 50-51 years will be used as the reference group, and never smokers will be used as the reference group for smoking exposures. The models will be adjusted for Body mass index, education level, ethnicity, number of children, and age at menarche. Data will be presented as relative risk ratio (RRR) and 95% CI.
3. Several sensitivity analyses and meta-analysis:
4. Since the UK Biobank data contributed more than 60% of the total sample used in the cross-sectional analyses, a sensitivity analysis will be conducted by excluding data from the UK Biobank study.
5. Another sensitivity analysis will be performed to analyse the effect of intensity, duration, age start of smoking and years since quitting smoking by adjusting covariates of non-smoking factors and smoking factors.
6. Meta-analysis: In both cross-sectional and prospective analyses, study-specific regressions and random-effects meta-analysis for the associations between each level of cigarette smoking measures and each category of menopausal age will be performed.
