# Supplementary material for: Relationships between intensity, duration, cumulative dose, and timing of smoking with age at menopause: A pooled analysis of individual data from 17 observational studies
Source: PLoS Med. 2018 Nov 27;15(11):e1002704. doi: 10.1371/journal.pmed.1002704 (PMC6258514; doi:10.1371/journal.pmed.1002704)
Supplement: S3 Text — (DOCX) [file pmed.1002704.s003.docx]

**S3 Text. Funding details of studies contributed to the InterLACE consortium**

ALSWH (including the data from the University of Newcastle and the University of Queensland) was supported by the Australian Government Department of Health. HOW was supported by the Queensland University of Technology Early Career Research Grant and the JSPS Grant-in-aid for Scientific Research. MCCS was supported by VicHealth and the Cancer Council, Victoria, Australia. DNCS was supported by the National Institute of Public Health, Copenhagen, Denmark. WLHS was funded by a grant from the Swedish Research Council (Grant number 521-2011-2955). NSHD has core funding from the UK Medical Research Council (MC UU 12019/1). NCDS is funded by the Economic and Social Research Council. ELSA is funded by the National Institute on Aging (Grants 2RO1AG7644 and 2RO1AG017644-01A1) and a consortium of UK government departments. UKWCS was funded by the World Cancer Research Fund. The Whitehall II study has been supported by grants from the Medical Research Council. SABRE study is supported by a joint programme grant from the Wellcome Trust and British Heart Foundation (BHF). HILO was supported by NIH grant 5 S06 GM08073-26. SMWHS was supported in part by grants from the National Institute of Nursing Research, P50-NU02323, P30-NR04001, and R01-NR0414. Baseline survey of the JNHS was supported in part by a Grant-in-Aid for Scientific Research (B: 14370133, 18390195) from the Japan Society for the Promotion of Science, and by the grants from the Japan Menopause Society. The Three-City Study is conducted under a partnership agreement between the Institut National de la Santé et de la Recherche Médicale (INSERM), the Victor Segalen-Bordeaux II University and Sanofi-Aventis. The Fondation pour la Recherche Médicale funded preparation and initiation of the study. The Three-City Study is also supported by the Caisse Nationale Maladie des Travailleurs Salariés, Direction Générale de la Santé, MGEN, Institut de la Longévité, Conseils Régionaux of Aquitaine and Bourgogne, Fondation de France, and Ministry of Research-INSERM Programme ‘Cohortes et collections de donneés biologiques’. This research has been conducted using the UK Biobank resource under application 26629.

SWAN has grant support from the National Institutes of Health (NIH), DHHS, through the National Institute on Aging (NIA), the National Institute of Nursing Research (NINR) and the NIH Office of Research on Women’s Health (ORWH) (Grants U01NR004061; U01AG012505, U01AG012535, U01AG012531, U01AG012539, U01AG012546, U01AG012553, U01AG012554, U01AG012495). Clinical Centers: *University of Michigan, Ann Arbor – Siobán Harlow, PI 2011 – present, MaryFran Sowers, PI 1994-2011*; *Massachusetts General Hospital, Boston, MA – Joel Finkelstein, PI 1999 – present*; *Robert Neer, PI 1994 – 1999; Rush University, Rush University Medical Center, Chicago, IL – Howard Kravitz, PI 2009 – present*; *Lynda Powell, PI 1994 – 2009; University of California, Davis/Kaiser – Ellen Gold, PI*; *University of California, Los Angeles – Gail Greendale, PI*; *Albert Einstein College of Medicine, Bronx, NY – Carol Derby, PI 2011 – present, Rachel Wildman, PI 2010 – 2011; Nanette Santoro, PI 2004 – 2010; University of Medicine and Dentistry – New Jersey Medical School, Newark – Gerson Weiss, PI 1994 – 2004;* and the *University of Pittsburgh, Pittsburgh, PA – Karen Matthews, PI.*

NIH Program Office: *National Institute on Aging, Bethesda, MD – Chhanda Dutta 2016 – present; Winifred Rossi 2012 – 2016; Sherry Sherman 1994 – 2012; Marcia Ory 1994 – 2001; National Institute of Nursing Research, Bethesda, MD – Program Officers.*

Central Laboratory: *University of Michigan, Ann Arbor – Daniel McConnell*  (Central Ligand Assay Satellite Services).

Coordinating Center: *University of Pittsburgh, Pittsburgh, PA – Maria Mori Brooks, PI 2012 - present; Kim Sutton-Tyrrell, PI* *2001 – 2012; New England Research Institutes, Watertown, MA - Sonja McKinlay, PI* *1995 – 2001.*

Steering Committee: Susan Johnson, Current Chair

Chris Gallagher, Former Chair

The content of this article is solely the responsibility of the authors and does not necessarily represent the official views of the NIA, NINR, ORWH or the NIH.
