# Supplementary material for: Relationships between intensity, duration, cumulative dose, and timing of smoking with age at menopause: A pooled analysis of individual data from 17 observational studies
Source: PLoS Med. 2018 Nov 27;15(11):e1002704. doi: 10.1371/journal.pmed.1002704 (PMC6258514; doi:10.1371/journal.pmed.1002704)
Supplement: S1 Table — (DOCX) [file pmed.1002704.s004.docx]

| **S1 Table.** Cross-sectional associations between cigarette smoking and age at menopause-further adjusted age at menarche and parity in 14 studies (n=194 368) ^*^ | | | | | | | | | | |
| --- | --- | --- | --- | --- | --- | --- | --- | --- | --- | --- |
|  | Age in years at menopause, n (%) | | | | |  | Adjusted RRR (95% CI) ^†^ | | | |
|  | <40 | 40-44 | 45-49 | 50-51 | ≥52 |  | <40 | 40-44 | 45-49 | ≥ 52 |
| Smoking status |  |  |  |  |  |  |  |  |  |  |
| Never smoker | 1834 (1.7) | 7222 (6.5) | 25901 (23.4) | 27353 (24.7) | 48382 (43.7) |  | 1.00 | 1.00 | 1.00 | 1.00 |
| Former smoker | 1118 (1.8) | 4497 (7.4) | 14975 (24.6) | 14661 (24.1) | 25641 (42.1) |  | 1.12 (1.01, 1.23) | 1.14 (1.03, 1.27) | 1.08 (1.05, 1.12) | 0.97 (0.94, 1) |
| Current smoker | 701 (3.1) | 2477 (10.9) | 7552 (33.1) | 5276 (23.2) | 6778 (29.7) |  | 1.94 (1.59, 2.36) | 1.77 (1.63, 1.92) | 1.45 (1.4, 1.51) | 0.75 (0.71, 0.79) |
| Intensity of smoking, cigarettes/day |  |  |  |  |  |  |  |  |  |  |
| Never smoker | 1834 (1.7) | 7222 (6.5) | 25901 (23.4) | 27353 (24.7) | 48382 (43.7) |  | 1.00 | 1.00 | 1.00 | 1.00 |
| Former smokers + 1-9 cigs/day | 111 (1.5) | 471 (6.2) | 1850 (24.4) | 1859 (24.5) | 3297 (43.5) |  | 0.93 (0.73, 1.2) | 0.98 (0.77, 1.26) | 1.03 (0.97, 1.08) | 1.02 (0.97, 1.07) |
| Former smokers + 10-19 cigs/day | 269 (1.9) | 1118 (7.8) | 3667 (25.4) | 3401 (23.6) | 5969 (41.4) |  | 1.17 (1.02, 1.35) | 1.23 (1.12, 1.34) | 1.16 (1.11, 1.2) | 0.96 (0.9, 1.04) |
| Former smokers + 20 or more cigs/day | 385 (2.4) | 1481 (9.1) | 4205 (25.7) | 3871 (23.7) | 6398 (39.2) |  | 1.37 (1.27, 1.47) | 1.37 (1.26, 1.5) | 1.16 (1.1, 1.22) | 0.9 (0.86, 0.93) |
| Current smokers + 1-9 cigs/day | 105 (2.4) | 382 (8.7) | 1377 (31.5) | 1040 (23.8) | 1474 (33.7) |  | 1.5 (1.18, 1.91) | 1.39 (1.16, 1.66) | 1.34 (1.26, 1.42) | 0.83 (0.78, 0.88) |
| Current smokers + 10-19 cigs/day | 267 (3.4) | 943 (11.9) | 2657 (33.7) | 1824 (23.1) | 2201 (27.9) |  | 2.12 (1.56, 2.88) | 1.92 (1.6, 2.3) | 1.48 (1.37, 1.6) | 0.7 (0.65, 0.76) |
| Current smokers + 20 or more cigs/day | 230 (4) | 736 (12.8) | 2019 (35.2) | 1251 (21.8) | 1502 (26.2) |  | 2.53 (2.11, 3.05) | 2.14 (1.92, 2.38) | 1.65 (1.5, 1.8) | 0.69 (0.64, 0.75) |
| Duration of smoking before menopause, years |  |  |  |  |  |  |  |  |  |  |
| Never smoker | 1834 (1.7) | 7222 (6.5) | 25901 (23.4) | 27353 (24.7) | 48382 (43.7) |  | 1.00 | 1.00 | 1.00 | 1.00 |
| Former smokers + duration <10 | 110 (1.6) | 433 (6.3) | 1639 (23.7) | 1701 (24.6) | 3044 (43.9) |  | 1.08 (0.92, 1.25) | 1.02 (0.93, 1.12) | 1.03 (0.92, 1.16) | 1 (0.95, 1.05) |
| Former smokers + duration 10-14 | 151 (2.3) | 411 (6.2) | 1625 (24.3) | 1663 (24.9) | 2829 (42.4) |  | 1.4 (0.95, 2.05) | 0.95 (0.75, 1.19) | 1.04 (0.99, 1.1) | 0.95 (0.86, 1.04) |
| Former smokers + duration 15-20 | 176 (3.2) | 477 (8.6) | 1284 (23.3) | 1286 (23.3) | 2298 (41.6) |  | 1.99 (1.39, 2.85) | 1.38 (1.22, 1.57) | 1.06 (1.02, 1.1) | 0.99 (0.95, 1.04) |
| Current smokers + duration <10 | 30 (9.8) | 45 (14.7) | 118 (38.4) | 48 (15.6) | 66 (21.5) |  | 9.83 (6.22, 15.54) | 3.64 (2.22, 5.95) | 2.59 (2.01, 3.35) | 0.79 (0.39, 1.58) |
| Current smokers + duration 10-14 | 103 (18.1) | 108 (19) | 170 (29.9) | 96 (16.9) | 91 (16) |  | 13.64 (9.34, 19.43) | 4.35 (2.9, 6.55) | 1.87 (1.65, 2.12) | 0.55 (0.46, 0.64) |
| Current smokers + duration 15-20 | 206 (19.3) | 260 (24.4) | 342 (32.1) | 157 (14.7) | 101 (9.5) |  | 16.71 (12.69, 21.7) | 6.49 (4.72, 8.93) | 2.28 (2.06, 2.52) | 0.37 (0.28, 0.5) |
| Cumulative dose of smoking, pack-years |  |  |  |  |  |  |  |  |  |  |
| Never smoker | 1834 (1.7) | 7222 (6.5) | 25901 (23.4) | 27353 (24.7) | 48382 (43.7) |  | 1.00 | 1.00 | 1.00 | 1.00 |
| Former smokers + pack-years ≤5 | 97 (1.4) | 406 (6) | 1576 (23.4) | 1648 (24.4) | 3015 (44.7) |  | 0.97 (0.71, 1.33) | 0.98 (0.79, 1.22) | 1.01 (0.92, 1.09) | 1.03 (0.95, 1.12) |
| Former smokers + pack-years 6-10 | 126 (2.1) | 407 (6.8) | 1400 (23.3) | 1466 (24.4) | 2621 (43.5) |  | 1.33 (1.01, 1.74) | 1.06 (0.95, 1.18) | 1.05 (0.98, 1.12) | 0.97 (0.95, 1) |
| Former smokers + pack-years 11-15 | 168 (2.3) | 580 (7.9) | 1896 (25.8) | 1755 (23.9) | 2957 (40.2) |  | 1.39 (0.8, 2.39) | 1.23 (0.88, 1.72) | 1.11 (1.01, 1.23) | 0.95 (0.91, 1) |
| Current smokers + pack-years <5 | 55 (4.8) | 134 (11.7) | 390 (34) | 257 (22.4) | 310 (27.1) |  | 3.46 (2.24, 5.34) | 2.06 (1.52, 2.81) | 1.56 (1.41, 1.74) | 0.68 (0.58, 0.81) |
| Current smokers + pack-years 6-10 | 96 (4.8) | 251 (12.4) | 655 (32.5) | 430 (21.3) | 586 (29) |  | 3.53 (2.74, 4.54) | 2.27 (1.74, 2.96) | 1.6 (1.45, 1.77) | 0.77 (0.64, 0.92) |
| Current smokers + pack-years 11-15 | 148 (5.7) | 419 (16) | 959 (36.7) | 538 (20.6) | 550 (21) |  | 4.2 (2.75, 6.4) | 3 (2.14, 4.22) | 1.85 (1.61, 2.13) | 0.58 (0.5, 0.69) |
| Age started smoking, years |  |  |  |  |  |  |  |  |  |  |
| Never smoker | 1834 (1.7) | 7222 (6.5) | 25901 (23.4) | 27353 (24.7) | 48382 (43.7) |  | 1.00 | 1.00 | 1.00 | 1.00 |
| Former smokers + age started at ≥20 | 176 (1.6) | 784 (7) | 2859 (25.6) | 2769 (24.7) | 4600 (41.1) |  | 0.95 (0.72, 1.27) | 1.08 (0.88, 1.33) | 1.08 (1.01, 1.16) | 0.95 (0.85, 1.05) |
| Former smokers + age started at 16-19 | 401 (1.8) | 1692 (7.7) | 5414 (24.6) | 5266 (23.9) | 9260 (42) |  | 1.12 (0.95, 1.33) | 1.2 (1.04, 1.39) | 1.1 (1.03, 1.17) | 0.97 (0.94, 1) |
| Former smokers + age started at ≤15 | 211 (2.5) | 761 (8.9) | 2371 (27.9) | 1978 (23.2) | 3189 (37.5) |  | 1.48 (1.26, 1.73) | 1.38 (1.21, 1.57) | 1.28 (1.17, 1.4) | 0.88 (0.84, 0.91) |
| Current smokers + age started at ≥20 | 178 (2.5) | 713 (10.1) | 2400 (33.9) | 1740 (24.6) | 2045 (28.9) |  | 1.61 (1.28, 2.03) | 1.62 (1.55, 1.7) | 1.44 (1.37, 1.52) | 0.69 (0.6, 0.79) |
| Current smokers + age started at 16-19 | 234 (3) | 882 (11.1) | 2667 (33.7) | 1834 (23.2) | 2302 (29.1) |  | 1.87 (1.58, 2.2) | 1.8 (1.59, 2.04) | 1.55 (1.42, 1.69) | 0.71 (0.69, 0.73) |
| Current smokers + age started at ≤ 15 | 196 (4.5) | 575 (13.1) | 1546 (35.3) | 905 (20.6) | 1161 (26.5) |  | 3.03 (2.04, 4.48) | 2.31 (1.95, 2.73) | 1.82 (1.66, 2) | 0.72 (0.66, 0.78) |
| Years since quitting smoking before menopause, years |  |  |  |  |  |  |  |  |  |  |
| Never smoker | 1834 (1.7) | 7222 (6.5) | 25901 (23.4) | 27353 (24.7) | 48382 (43.7) |  | 1.00 | 1.00 | 1.00 | 1.00 |
| Current smoker | 701 (3.1) | 2477 (10.9) | 7552 (33.1) | 5276 (23.2) | 6778 (29.7) |  | 1.93 (1.56, 2.4) | 1.75 (1.61, 1.91) | 1.49 (1.38, 1.62) | 0.75 (0.71, 0.79) |
| 1-5 | 212 (3.1) | 868 (12.8) | 2205 (32.4) | 1845 (27.1) | 1671 (24.6) |  | 1.63 (1.53, 1.74) | 1.72 (1.51, 1.96) | 1.45 (1.39, 1.51) | 0.5 (0.47, 0.54) |
| 6-10 | 171 (2.8) | 741 (12.3) | 1883 (31.2) | 1813 (30.0) | 1428 (23.7) |  | 1.35 (0.99, 1.83) | 1.51 (1.36, 1.67) | 1.27 (1.18, 1.36) | 0.44 (0.4, 0.48) |
| 11-15 | 140 (1.8) | 810 (10.6) | 2410 (31.6) | 2467 (32.4) | 1794 (23.5) |  | 0.83 (0.74, 0.94) | 1.23 (1.06, 1.42) | 1.03 (0.97, 1.09) | 0.41 (0.39, 0.43) |
| ^*^ Three studies (WHITEHALL, SABRE and HOW) were excluded for no information on age at menarche or parity.  ^†^ Multinomial logistic regression model was used to estimate relative risk ratio (RRR) and 95% confidence interval (95% CI) with the category of 50-51 years as reference. All RRRs were adjusted for race/ethnicity/region, education level, and body mass index. | | | | | | | | | | |
