# Supplementary material for: Relationships between intensity, duration, cumulative dose, and timing of smoking with age at menopause: A pooled analysis of individual data from 17 observational studies
Source: PLoS Med. 2018 Nov 27;15(11):e1002704. doi: 10.1371/journal.pmed.1002704 (PMC6258514; doi:10.1371/journal.pmed.1002704)
Supplement: S2 Table — (DOCX) [file pmed.1002704.s005.docx]

| **S2 Table.** Cross-sectional associations between cigarette smoking and age at menopause after excluding UK Biobank study (n=69 217) | | | | | | | | | | |
| --- | --- | --- | --- | --- | --- | --- | --- | --- | --- | --- |
|  | Age in years at menopause, n (%) | | | | |  | Adjusted RRR (95% CI) ^*^ | | | |
|  | <40 | 40-44 | 45-49 | 50-51 | ≥52 |  | <40 | 40-44 | 45-49 | ≥ 52 |
| Smoking status | | |  |  |  |  |  |  |  |  |
| Never smoker | 662 (1.7) | 2524 (6.5) | 9866 (25.5) | 9862 (25.5) | 15730 (40.7) |  | 1.00 | 1.00 | 1.00 | 1.00 |
| Former smoker | 305 (1.8) | 1154 (6.6) | 4756 (27.3) | 4395 (25.2) | 6805 (39.1) |  | 1.04 (0.86, 1.26) | 1.01 (0.87, 1.18) | 1.06 (0.97, 1.17) | 0.95 (0.90, 1.01) |
| Current smoker | 362 (2.8) | 1346 (10.2) | 4640 (35.3) | 3103 (23.6) | 3707 (28.2) |  | 1.78 (1.47, 2.16) | 1.69 (1.52, 1.89) | 1.46 (1.33, 1.61) | 0.75 (0.68, 0.82) |
| Intensity of smoking, cigarettes/day | | |  |  |  |  |  |  |  |  |
| Never smoker | 662 (1.7) | 2524 (6.5) | 9866 (25.5) | 9862 (25.5) | 15730 (40.7) |  | 1.00 | 1.00 | 1.00 | 1.00 |
| Former smokers + 1-9 cigs/day | 57 (1.3) | 235 (5.4) | 1146 (26.5) | 1105 (25.5) | 1789 (41.3) |  | 0.78 (0.58, 1.06) | 0.81 (0.69, 0.96) | 1.01 (0.89, 1.15) | 0.99 (0.93, 1.05) |
| Former smokers + 10-19 cigs/day | 55 (1.7) | 237 (7.3) | 959 (29.4) | 828 (25.4) | 1186 (36.3) |  | 1.02 (0.70, 1.48) | 1.11 (0.84, 1.45) | 1.14 (0.99, 1.30) | 0.87 (0.72, 1.04) |
| Former smokers + 20 or more cigs/day | 78 (2.5) | 259 (8.4) | 846 (27.5) | 771 (25.1) | 1119 (36.4) |  | 1.45 (1.01, 2.09) | 1.26 (0.95, 1.67) | 1.11 (0.96, 1.28) | 0.86 (0.74, 0.99) |
| Current smokers + 1-9 cigs/day | 57 (2.2) | 193 (7.6) | 830 (32.7) | 608 (24.0) | 847 (33.4) |  | 1.42 (0.95, 2.11) | 1.21 (1.03, 1.43) | 1.32 (1.18, 1.48) | 0.86 (0.78, 0.94) |
| Current smokers + 10-19 cigs/day | 121 (2.8) | 458 (10.6) | 1558 (36.1) | 1030 (23.9) | 1148 (26.6) |  | 1.74 (1.37, 2.22) | 1.68 (1.47, 1.91) | 1.46 (1.28, 1.66) | 0.69 (0.62, 0.77) |
| Current smokers + 20 or more cigs/day | 130 (3.6) | 458 (12.8) | 1283 (35.9) | 788 (22.0) | 916 (25.6) |  | 2.34 (1.91, 2.85) | 2.16 (1.86, 2.51) | 1.63 (1.37, 1.93) | 0.70 (0.62, 0.79) |
| Duration of smoking ^†^, years |  |  |  |  |  |  |  |  |  |  |
| Never smoker | 662 (1.7) | 2524 (6.5) | 9866 (25.5) | 9862 (25.5) | 15730 (40.7) |  | 1.00 | 1.00 | 1.00 | 1.00 |
| Former smokers + duration <10 | 40 (1.4) | 167 (5.8) | 768 (26.8) | 696 (24.3) | 1191 (41.6) |  | 0.73 (0.42, 1.28) | 0.89 (0.70, 1.13) | 0.99 (0.89, 1.1) | 1.07 (0.96, 1.19) |
| Former smokers + duration 10-20 | 45 (1.3) | 201 (5.9) | 947 (27.7) | 905 (26.4) | 1325 (38.7) |  | 0.86 (0.56, 1.31) | 0.99 (0.80, 1.23) | 1.04 (0.86, 1.25) | 0.90 (0.81, 1.00) |
| Current smokers + duration <10 | 17 (8.4) | 30 (14.8) | 82 (40.4) | 41 (20.2) | 33 (16.3) |  | 7.24 (3.28, 15.97) | 2.82 (1.48, 5.36) | 2.11 (1.71, 2.61) | 0.54 (0.31, 0.95) |
| Current smokers + duration 10-20 | 164 (14) | 257 (22) | 402 (34.4) | 199 (17) | 146 (12.5) |  | 14.58 (9.18, 23.15) | 5.35 (4.33, 6.62) | 2.00 (1.77, 2.26) | 0.47 (0.39, 0.55) |
| Cumulative quality of smoking, pack-years |  |  |  |  |  |  |  |  |  |  |
| Never smoker | 662 (1.7) | 2524 (6.5) | 9866 (25.5) | 9862 (25.5) | 15730 (40.7) |  | 1.00 | 1.00 | 1.00 | 1.00 |
| Former smokers + pack-years ≤5 | 45 (1.0) | 226 (5.2) | 1092 (25.4) | 1096 (25.5) | 1847 (42.9) |  | 0.61 (0.33, 1.14) | 0.83 (0.68, 1.01) | 0.97 (0.85, 1.1) | 1.03 (0.94, 1.13) |
| Former smokers + pack-years 6-10 | 30 (1.0) | 161 (5.4) | 871 (29.0) | 760 (25.3) | 1185 (39.4) |  | 0.67 (0.56, 0.80) | 0.83 (0.65, 1.07) | 1.10 (0.99, 1.22) | 0.96 (0.89, 1.03) |
| Former smokers + pack-years 11-15 | 39 (1.5) | 191 (7.2) | 774 (29.2) | 635 (24.0) | 1010 (38.1) |  | 0.88 (0.53, 1.49) | 1.15 (0.84, 1.56) | 1.19 (1.03, 1.36) | 0.97 (0.88, 1.07) |
| Current smokers + pack-years <5 | 25 (3.3) | 75 (9.9) | 280 (37.1) | 176 (23.3) | 199 (26.4) |  | 2.22 (1.56, 3.17) | 1.64 (1.28, 2.12) | 1.55 (1.34, 1.8) | 0.69 (0.57, 0.82) |
| Current smokers + pack-years 6-10 | 45 (4.3) | 127 (12.0) | 335 (31.8) | 218 (20.7) | 330 (31.3) |  | 3.31 (2.19, 5.02) | 2.21 (1.82, 2.69) | 1.49 (1.28, 1.72) | 0.93 (0.77, 1.13) |
| Current smokers + pack-years 11-15 | 57 (4.0) | 184 (12.9) | 548 (38.5) | 325 (22.8) | 311 (21.8) |  | 2.80 (1.79, 4.36) | 2.30 (1.56, 3.41) | 1.63 (1.49, 1.78) | 0.59 (0.49, 0.70) |
| Age started smoking, years | |  |  |  |  |  |  |  |  |  |
| Never smoker | 662 (1.7) | 2524 (6.5) | 9866 (25.5) | 9862 (25.5) | 15730 (40.7) |  | 1.00 | 1.00 | 1.00 | 1.00 |
| Former smokers + age started at ≥20 | 63 (1.3) | 292 (5.8) | 1399 (28.0) | 1338 (26.8) | 1900 (38.1) |  | 0.79 (0.45, 1.38) | 0.89 (0.70, 1.13) | 1.02 (0.91, 1.14) | 0.89 (0.82, 0.97) |
| Former smokers + age started at 16-19 | 95 (1.5) | 386 (6.2) | 1651 (26.4) | 1589 (25.4) | 2525 (40.4) |  | 0.91 (0.68, 1.22) | 0.94 (0.85, 1.04) | 1.03 (0.91, 1.17) | 0.96 (0.90, 1.03) |
| Former smokers + age started at ≤15 | 53 (2.1) | 200 (7.8) | 765 (29.8) | 602 (23.5) | 944 (36.8) |  | 1.25 (0.92, 1.68) | 1.25 (0.87, 1.78) | 1.27 (1.01, 1.59) | 0.95 (0.87, 1.04) |
| Current smokers + age started at ≥20 | 113 (2.2) | 492 (9.5) | 1852 (35.9) | 1296 (25.2) | 1400 (27.2) |  | 1.50 (1.08, 2.08) | 1.57 (1.41, 1.75) | 1.38 (1.24, 1.53) | 0.69 (0.64, 0.75) |
| Current smokers + age started at 16-19 | 122 (2.8) | 455 (10.5) | 1519 (34.9) | 1004 (23.1) | 1251 (28.8) |  | 1.82 (1.43, 2.31) | 1.74 (1.42, 2.13) | 1.49 (1.31, 1.70) | 0.77 (0.71, 0.82) |
| Current smokers + age started at ≤ 15 | 77 (3.4) | 273 (11.9) | 856 (37.3) | 492 (21.4) | 598 (26.0) |  | 2.16 (1.50, 3.10) | 2.05 (1.75, 2.41) | 1.73 (1.45, 2.08) | 0.74 (0.65, 0.85) |
| Years since quitting smoking, years | | |  |  |  |  |  |  |  |  |
| Never smoker | 662 (1.7) | 2524 (6.5) | 9866 (25.5) | 9862 (25.5) | 15730 (40.7) |  | 1.00 | 1.00 | 1.00 | 1.00 |
| Current smoker | 362 (2.8) | 1346 (10.2) | 4640 (35.3) | 3103 (23.6) | 3707 (28.2) |  | 1.78 (1.46, 2.17) | 1.70 (1.52, 1.90) | 1.47 (1.33, 1.61) | 0.75 (0.68, 0.82) |
| 1-5 | 61 (2.9) | 212 (10.2) | 819 (39.3) | 590 (28.3) | 400 (19.2) |  | 1.66 (1.15, 2.41) | 1.44 (1.18, 1.75) | 1.47 (1.21, 1.78) | 0.42 (0.36, 0.49) |
| 6-10 | 28 (1.8) | 164 (10.4) | 656 (41.7) | 436 (27.7) | 289 (18.4) |  | 1.03 (0.36, 2.94) | 1.51 (1.11, 2.06) | 1.35 (1.22, 1.50) | 0.41 (0.36, 0.46) |
| 11-15 | 38 (1.7) | 198 (8.7) | 810 (35.7) | 736 (32.4) | 489 (21.5) |  | 0.85 (0.57, 1.28) | 1.11 (0.80, 1.54) | 1.07 (0.87, 1.31) | 0.41 (0.36, 0.47) |
| ^*^ Multinomial logistic regression model was used to estimate relative risk ratio (RRR) and 95% confidence interval (95% CI) with the category of 50-51 years as reference. All RRRs were adjusted for race/ethnicity/region, education level, and body mass index.  ^†^ The categories of 10-14 and 15-20 were combined as 10-20 for analysis for limited number of women with premature menopause.  Abbreviations: cigs, cigarettes. | | | | | | | | | | |
