# Supplementary material for: Relationships between intensity, duration, cumulative dose, and timing of smoking with age at menopause: A pooled analysis of individual data from 17 observational studies
Source: PLoS Med. 2018 Nov 27;15(11):e1002704. doi: 10.1371/journal.pmed.1002704 (PMC6258514; doi:10.1371/journal.pmed.1002704)
Supplement: S3 Table — (DOCX) [file pmed.1002704.s006.docx]

| **S3 Table.** Cross-sectional association between intensity, duration, age started smoking and age at menopause-adjusted other smoking factors | | | | |
| --- | --- | --- | --- | --- |
|  | Adjusted: Non-smoking factors + Smoking factors^*^ | | | |
|  | <40 | 40-44 | 45-49 | ≥ 52 |
| Intensity of smoking, cigarettes/day | | |  |  |
| Former smokers + 1-9 cigs/day^†^ | 1.00 | 1.00 | 1.00 | 1.00 |
| Former smokers + 10-19 cigs/day | 1.25 (0.96, 1.63) | 1.23 (1.05, 1.44) | 1.08 (1.04, 1.12) | 0.97 (0.92, 1.03) |
| Former smokers + 20 or more cigs/day | 1.51 (1.14, 1.99) | 1.42 (1.22, 1.65) | 1.09 (1.03, 1.15) | 0.90 (0.84, 0.96) |
| Current smokers + 1-9 cigs/day | 1.70 (1.41, 2.07) | 1.42 (1.28, 1.57) | 1.32 (1.23, 1.41) | 0.82 (0.75, 0.90) |
| Current smokers + 10-19 cigs/day | 2.37 (1.93, 2.90) | 1.95 (1.78, 2.14) | 1.47 (1.38, 1.57) | 0.69 (0.65, 0.74) |
| Current smokers + 20 or more cigs/day | 2.88 (2.46, 3.38) | 2.22 (1.78, 2.78) | 1.61 (1.45, 1.79) | 0.66 (0.61, 0.73) |
| Duration of smoking before menopause, years | | |  |  |
| Former smokers + duration <10^†^ | 1.00 | 1.00 | 1.00 | 1.00 |
| Former smokers + duration 10-14 | 1.48 (1.18, 1.86) | 0.95 (0.81, 1.12) | 1.09 (0.99, 1.20) | 0.95 (0.88, 1.02) |
| Former smokers + duration 15-20 | 2.15 (1.78, 2.61) | 1.36 (1.21, 1.53) | 1.09 (1.00, 1.19) | 0.96 (0.90, 1.03) |
| Current smokers + duration <10 | 12.94 (8.84, 18.93) | 4.24 (2.29, 7.86) | 2.96 (2.15, 4.09) | 0.77 (0.38, 1.56) |
| Current smokers + duration 10-14 | 14.72 (9.12, 21.96) | 5.45 (3.43, 8.68) | 2.21 (1.83, 2.67) | 0.53 (0.41, 0.70) |
| Current smokers + duration 15-20 | 15.62 (7.81, 31.26) | 7.78 (5.97, 10.15) | 2.41 (2.12, 2.75) | 0.37 (0.26, 0.51) |
| Age started smoking, years | |  |  |  |
| Former smokers + age initiated at ≥20^†^ | 1.00 | 1.00 | 1.00 | 1.00 |
| Former smokers + age initiated at 16-19 | 1.09 (0.91, 1.30) | 1.07 (0.97, 1.18) | 1.00 (0.95, 1.06) | 1.03 (0.96, 1.11) |
| Former smokers + age initiated at ≤15 | 1.42 (1.18, 1.72) | 1.23 (1.05, 1.43) | 1.16 (1.03, 1.32) | 0.94 (0.84, 1.04) |
| Current smokers + age initiated at ≥20 | 1.64 (1.40, 1.91) | 1.49 (1.27, 1.76) | 1.33 (1.25, 1.40) | 0.74 (0.69, 0.80) |
| Current smokers + age initiated at 16-19 | 1.95 (1.53, 2.47) | 1.63 (1.35, 1.98) | 1.43 (1.34, 1.53) | 0.76 (0.67, 0.85) |
| Current smokers + age initiated at ≤ 15 | 2.92 (2.38, 3.60) | 2.04 (1.80, 2.31) | 1.65 (1.47, 1.85) | 0.74 (0.68, 0.81) |
| Years since quitting smoking, years | | |  |  |
| Current smoker | 2.59 (1.36, 4.93) | 1.28 (0.91, 1.81) | 1.45 (1.19, 1.77) | 0.71 (0.62, 0.81) |
| 1-5 | 2.08 (1.19, 3.63) | 1.17 (0.92, 1.50) | 1.35 (1.09, 1.69) | 0.75 (0.66, 0.85) |
| 6-10 | 1.58 (1.19, 2.11) | 1.18 (1.01, 1.39) | 1.17 (1.05, 1.31) | 0.8 (0.72, 0.90) |
| 11-15^†^ | 1.00 | 1.00 | 1.00 | 1.00 |
| ^*^ Multinomial logistic regression model was used to estimate relative risk ratio (RRR) and 95% confidence interval (95% CI) with the category of 50-51 years as reference. All RRRs were adjusted for race/ethnicity/region, education level, and body mass index. Further, intensity and duration of smoking were adjusted for each other; age started smoking and years since quitting smoking were adjusted for pack-years.  ^†^ The lowest level in former smokers was used as reference group.  Abbreviations: cigs, cigarettes; RRR, relative risk ratio. | | | | |
