# Supplementary material for: Relationships between intensity, duration, cumulative dose, and timing of smoking with age at menopause: A pooled analysis of individual data from 17 observational studies
Source: PLoS Med. 2018 Nov 27;15(11):e1002704. doi: 10.1371/journal.pmed.1002704 (PMC6258514; doi:10.1371/journal.pmed.1002704)
Supplement: S4 Table — (DOCX) [file pmed.1002704.s007.docx]

| **S4 Table.** Meta-analysis results from study-specific cross-sectional associations between cigarette smoking and age at menopause | | | | |
| --- | --- | --- | --- | --- |
|  | Adjusted RRR (95% CI)^*^ | | | |
|  | <40 years | 40-44 years | 45-49 years | ≥52 years |
| Smoking status | | |  |  |
| Never smoker | 1.00 | 1.00 | 1.00 | 1.00 |
| Former smoker | 1.15 (1.07, 1.25) | 1.14 (1.04, 1.25) | 1.09 (1.03, 1.14) | 0.97 (0.90, 1.03) |
| Current smoker | 2.22 (2.02, 2.44) | 1.84 (1.73, 1.96) | 1.45 (1.39, 1.52) | 0.75 (0.70, 0.81) |
| Intensity of smoking, cigarettes/day | | | | |
| Never smoker | 1.00 | 1.00 | 1.00 | 1.00 |
| Former smokers + 1-9 cigs/day | 0.99 (0.81, 1.20) | 0.93 (0.77, 1.12) | 1.00 (0.91, 1.10) | 1.02 (0.96, 1.08) |
| Former smokers + 10-19 cigs/day | 1.22 (0.90, 1.65) | 1.20 (0.97, 1.48) | 1.15 (1.09, 1.21) | 0.89 (0.76, 1.04) |
| Former smokers + 20 or more cigs/day | 1.67 (1.14, 2.44)^†^ | 1.40 (1.19, 1.64) | 1.16 (1.09, 1.24) | 0.84 (0.74, 0.96) |
| Current smokers + 1-9 cigs/day | 1.76 (1.35, 2.29) | 1.44 (1.27, 1.63) | 1.32 (1.19, 1.46) | 0.85 (0.77, 0.94) |
| Current smokers + 10-19 cigs/day | 2.53 (2.18, 2.92) | 2.05 (1.88, 2.24) | 1.46 (1.32, 1.61) | 0.72 (0.65, 0.80) |
| Current smokers + 20 or more cigs/day | 2.87 (2.40, 3.43) | 2.18 (1.98, 2.40) | 1.58 (1.46, 1.71) | 0.66 (0.61, 0.71) |
| Duration of smoking, years | | | |  |
| Never smoker | 1.00 | 1.00 | 1.00 | 1.00 |
| Former smokers + duration <10 | 0.89 (0.61, 1.30)^†^ | 0.95 (0.79, 1.13) ^†^ | 0.98 (0.91, 1.07) | 1.06 (0.97, 1.15) |
| Former smokers + duration 10-14 | 1.10 (0.39, 3.15) ^†^ | 1.10 (0.57, 2.13) ^†^ | 0.94 (0.86, 1.03) | 0.95 (0.82, 1.09) |
| Former smokers + duration 15-20 | 1.13 (0.74, 1.73) | 1.29 (0.83, 2.01) | 1.07 (0.57, 2.02) | 0.94 (0.89, 0.98) |
| Current smokers + duration <10 | 11.03 (4.01, 30.35) | 2.74 (1.05, 7.11) ^†^ | 2.35 (2.21, 2.49) | 0.55 (0.27, 1.08) |
| Current smokers + duration 10-14 | 8.47 (3.69, 19.42) ^†^ | 3.36 (2.07, 5.45) | 2.06 (0.92, 4.64) ^†^ | 0.51 (0.42, 0.62) |
| Current smokers + duration 15-20 | 16.82 (8.00, 35.36) ^†^ | 6.96 (5.52, 8.76) | 2.11 (1.74, 2.56) | 0.42 (0.33, 0.54) |
| Cumulative dose of smoking, pack-years | | |  |  |
| Never smoker | 1.00 | 1.00 | 1.00 | 1.00 |
| Former smokers + pack years ≤5 | 0.87 (0.41, 1.87) ^†^ | 0.94 (0.72, 1.22) | 0.96 (0.79, 1.17) ^†^ | 1.03 (0.91, 1.17) |
| Former smokers + pack years 6-10 | 1.04 (0.84, 1.28) | 0.97 (0.79, 1.19) | 0.99 (0.93, 1.07) | 1.07 (0.97, 1.18) |
| Former smokers + pack years 11-15 | 1.05 (0.51, 2.17) ^†^ | 1.11 (0.77, 1.61) ^†^ | 1.15 (1.08, 1.23) | 0.94 (0.89, 1.00) |
| Current smokers + pack years ≤5 | 3.74 (2.41, 5.81) | 1.91 (1.51, 2.40) | 1.44 (1.22, 1.69) | 0.74 (0.63, 0.88) |
| Current smokers + pack years 6-10 | 3.74 (2.67, 5.23) | 2.26 (1.92, 2.66) | 1.51 (1.33, 1.71) | 0.88 (0.69, 1.14) |
| Current smokers + pack years 11-15 | 4.30 (3.19, 5.78) | 2.59 (1.89, 3.55) ^†^ | 1.69 (1.48, 1.94) | 0.64 (0.57, 0.73) |
| Age started smoking, years | | | |  |
| Never smoker | 1.00 | 1.00 | 1.00 | 1.00 |
| Former smokers + age started at ≥20 | 1.04 (0.76, 1.43) | 1.00 (0.80, 1.25)^†^ | 1.03 (0.95, 1.12) | 0.95 (0.89, 1.02) |
| Former smokers + age started at 16-19 | 1.14 (0.94, 1.39) | 1.14 (1.02, 1.27) | 1.06 (0.97, 1.15) | 0.94 (0.88, 1.01) |
| Former smokers + age started at ≤15 | 1.60 (1.37, 1.86) | 1.41 (1.13, 1.75)^†^ | 1.21 (1.07, 1.38) | 0.88 (0.83, 0.93) |
| Current smokers + age started at ≥20 | 1.96 (1.65, 2.32) | 1.68 (1.52, 1.85) | 1.34 (1.25, 1.44) | 0.75 (0.69, 0.81) |
| Current smokers + age started at 16-19 | 2.26 (1.88, 2.72) | 1.97 (1.73, 2.26) | 1.50 (1.41, 1.60) | 0.74 (0.68, 0.80) |
| Current smokers + age started at ≤15 | 3.14 (2.47, 4.00) | 2.50 (2.25, 2.79) | 1.72 (1.55, 1.90) | 0.71 (0.65, 0.78) |
| Years since quitting smoking, years | | | | |
| Never smoker | 1.00 | 1.00 | 1.00 | 1.00 |
| Current smoker | 2.32 (2.10, 2.55) | 1.90 (1.79, 2.01) | 1.46 (1.39, 1.53) | 0.72 (0.67, 0.78) |
| 1-5 | 2.03 (1.32, 3.12) | 1.69 (1.30, 2.19) | 1.51 (1.39, 1.65) | 1.02 (0.93, 1.12) |
| 6-10 | 1.24 (0.77, 2.00) | 1.54 (1.36, 1.75) | 1.33 (1.21, 1.47) | 0.89 (0.82, 0.97) |
| 11-15 | 0.90 (0.70, 1.15) | 1.17 (0.90, 1.52) | 1.04 (0.92, 1.18) | 0.82 (0.76, 0.89) |
| ^*^ In each study, multinomial logistic regression model was used to estimate relative risk ratio (RRR) and 95% confidence interval (95% CI) with the category of 50-51 years as reference, and race/ethnicity/region, education level, and body mass index were included in all models. The estimates from each study were combined using random-effects meta-analysis for each category of menopause age.  ^†^ Significant heterogeneity between studies (*P* <0.05). | | | | |
| Abbreviations: cigs, cigarettes; RRR, relative risk ratio. | | | | |
