# Supplementary material for: Relationships between intensity, duration, cumulative dose, and timing of smoking with age at menopause: A pooled analysis of individual data from 17 observational studies
Source: PLoS Med. 2018 Nov 27;15(11):e1002704. doi: 10.1371/journal.pmed.1002704 (PMC6258514; doi:10.1371/journal.pmed.1002704)
Supplement: S5 Table — (DOCX) [file pmed.1002704.s008.docx]

| **S5 Table.** Meta-analysis results from study specific prospective associations between cigarette smoking and age at menopause | | | |
| --- | --- | --- | --- |
|  | RRR (95% CI) ^*^ | | |
|  | <45 years | 45-49 years | ≥52 years |
| Smoking status |  |  |  |
| Never smoker | 1.00 | 1.00 | 1.00 |
| Former smoker | 1.23 (0.98, 1.54) | 1.11 (0.97, 1.27) | 1.04 (0.89, 1.21)^†^ |
| Current smoker | 1.83 (1.44, 2.33) | 1.60 (1.44, 1.78) | 0.80 (0.70, 0.91) |
| Intensity of smoking, cigarettes/day |  |  |  |
| Never smoker | 1.00 | 1.00 | 1.00 |
| Former smokers + 1-9 cigs/day | 1.01 (0.66, 1.57) | 0.94 (0.70, 1.27)^†^ | 0.97 (0.86, 1.09) |
| Former smokers + 10-19 cigs/day | 1.64 (0.71, 3.82) | 1.08 (0.88, 1.31) | 0.94 (0.69, 1.30)^†^ |
| Former smokers + 20 or more cigs/day | 2.01 (1.31, 3.08) | 1.31 (1.02, 1.68) | 0.82 (0.66, 1.02)^†^ |
| Current smokers + 1-9 cigs/day | 1.58 (0.91, 2.75) | 1.34 (1.07, 1.68) | 0.77 (0.64, 0.92) |
| Current smokers + 10-19 cigs/day | 2.03 (1.32, 3.12) | 1.71 (1.45, 2.03) | 0.80 (0.62, 1.03) |
| Current smokers + 20 or more cigs/day | 2.30 (1.53, 3.45) | 1.73 (1.44, 2.08) | 0.70 (0.60, 0.82) |
| Duration of smoking, years |  |  |  |
| Never smoker | 1.00 | 1.00 | 1.00 |
| Former smokers + duration <10 | 1.02 (0.61, 1.70) | 0.99 (0.87, 1.14) | 0.99 (0.89, 1.10) |
| Former smokers + duration 10-20 | 1.03 (0.70, 1.50) | 1.10 (0.92, 1.32) | 0.94 (0.74, 1.19) |
| Current smokers + duration <10 | 7.20 (3.37, 17.66) | 2.77 (1.33, 5.76) | 0.58 (0.29, 1.20) |
| Current smokers + duration 10-20 | 11.11 (5.14, 24.02) | 3.68 (2.66, 5.10) | 0.32 (0.23, 0.45) |
| Cumulative dose of smoking, pack-years |  |  |  |
| Never smoker | 1.00 | 1.00 | 1.00 |
| Former smokers + pack years ≤10 | 0.90 (0.57, 1.40) | 0.95 (0.80, 1.13) | 1.04 (0.86, 1.27) |
| Former smokers + pack years 11-15 | 1.29 (0.79, 2.10) | 1.14 (0.98, 1.33) | 0.96 (0.84, 1.09) |
| Current smokers + pack years ≤10 | 1.49 (0.90, 2.47) | 1.35 (0.97, 1.88) | 0.72 (0.50, 1.05) |
| Current smokers + pack years 11-15 | 2.47 (0.84, 7.29) | 1.79 (1.42, 2.27) | 0.91 (0.67, 1.24) |
| Age started smoking, years |  |  |  |
| Never smoker | 1.00 | 1.00 | 1.00 |
| Former smokers + age started at ≥20 | 0.71 (0.40, 1.26) | 1.02 (0.85, 1.23) | 0.94 (0.82, 1.08) |
| Former smokers + age started at 16-19 | 0.89 (0.64, 1.24) | 0.99 (0.86, 1.15) | 0.95 (0.83, 1.08) |
| Former smokers + age started at ≤15 | 2.79 (2.06, 3.78) | 1.27 (0.96, 1.70)^†^ | 0.86 (0.76, 0.98) |
| Current smokers + age started at ≥20 | 1.72 (0.73, 4.05) | 1.41 (1.18, 1.69) | 0.83 (0.66, 1.05) |
| Current smokers + age started at 16-19 | 1.61 (1.15, 2.27) | 1.42 (1.24, 1.63) | 0.72 (0.62, 0.83) |
| Current smokers + age started at ≤15 | 2.83 (2.01, 3.98) | 2.00 (1.70, 2.36) | 0.75 (0.64, 0.87) |
| Years since quitting smoking, years |  |  |  |
| Never smoker | 1.00 | 1.00 | 1.00 |
| Current smoker | 2.15 (1.54, 3.02) | 1.64 (1.48, 1.82) | 0.76 (0.68, 0.85) |
| 1-5 | 1.53 (1.01, 2.32) | 1.52 (1.15, 2.01) | 0.89 (0.72, 1.08) |
| 6-15 | 1.20 (0.66, 2.20) | 1.49 (1.19, 1.86) | 1.00 (0.85, 1.16) |
| 15+ | 0.69 (0.43, 1.09) | 0.85 (0.74, 0.98) | 1.00 (0.91, 1.09) |
| ^*^ In each study, multinomial logistic regression model was used to estimate relative risk ratio (RRR) and 95% confidence interval (95% CI) with the category of 50-51 years as reference, and race/ethnicity/region, education level, and body mass index were included in all models. The estimates from each study were combined using random-effects meta-analysis for each category of menopause age.  ^†^ Significant heterogeneity between studies (*P* <0.05). | | | |
| Abbreviations: cigs, cigarettes; RRR, relative risk ratio. | | | |
