# Supplementary material for: Relationships between intensity, duration, cumulative dose, and timing of smoking with age at menopause: A pooled analysis of individual data from 17 observational studies
Source: PLoS Med. 2018 Nov 27;15(11):e1002704. doi: 10.1371/journal.pmed.1002704 (PMC6258514; doi:10.1371/journal.pmed.1002704)
Supplement: S6 Table — (DOCX) [file pmed.1002704.s009.docx]

| S6 Table. List of contacts for data access | |
| --- | --- |
| **Study** | **Contact** |
| Australian Longitudinal Study on Women’s Health (ALSWH) | Contact ALSWH data at https://www.alswh.org.au/ Email: sph-wha@sph.uq.edu.au |
| Healthy Ageing of Women Study (HOW) | Contact with Charrlotte Seib, email: c.seib@griffith.edu.au |
| Melbourne Collaborative Cohort Study (MCCS) | Contact MCCS data at https://www.cancervic.org.au/research/epidemiology/health_2020/health2020-overview Email: Health2020@cancervic.org.au |
| Danish Nurse Cohort Study (DNC) | Contact with Yrsa A Hundrup, email: yrsand01@glo.regionh.dk |
| French Three-City Study (French 3C) | Contact French 3C data at http://www.three-city-study.com/ Email: E3C.U708@inserm.fr |
| Japan Nurses’ Health Study (JNHS) | Contact JNHS data at http://plaza.umin.ac.jp/~jnhs/e_index.html Email: jnhs.jimu@gmail.com |
| Women’s Lifestyle and Health Study (WLH) | Contact WLH data at https://ki.se/en/meb/womens-lifestyle-and-health Email: Elisabete.Weiderpass.Vainio@ki.se |
| MRC National Survey of Health and Development (NSHD) | Contact NSHD data at http://www.nshd.mrc.ac.uk/ Email: mrclha.enquiries@ucl.ac.uk |
| National Child Development Study (NCDS) | Contact NCDS data at http://www.cls.ioe.ac.uk/default.aspx Email: clsfeedback@ioe.ac.uk |
| English Longitudinal Study of Ageing (ELSA) | Contact ELSA data at https://www.elsa-project.ac.uk/ Email: ELSAdata@natcen.ac.uk. |
| UK Women's Cohort Study (UKWCS) | Contact UKWCS data at https://ukwcs.leeds.ac.uk/ Email: UKWCS@leeds.ac.uk |
| Whitehall II study (WHITEHALL) | Contact WHITEHALL II data at http://www.ucl.ac.uk/iehc/research/epidemiology-public-health/research/whitehallII Email: whitehall2@ucl.ac.uk |
| Southall And Brent REvisited (SABRE) | Contact SABRE data at https://www.sabrestudy.org/?cat=10 Email: sabre@ucl.ac.uk |
| UK Biobank (UK Biobank) | Contact UK Biobank data at http://www.ukbiobank.ac.uk/ Email: access@ukbiobank.ac.uk |
| Hilo Women's Healthy Study (HILO) | Contact with Lynn Morrison, email: lmorriso@hawaii.edu |
| Study of Women's Health Across the Nation (SWAN) | Contact SWAN data at https://www.swanstudy.org/ Email: swanaccess@edc.pitt.edu |
| Seattle Middle Women's Health Study (SMWHS) | Contact with Jerald R.Herting, email: herting@uw.edu |
